# Combining Genome-Scale Experimental and Computational Methods To Identify Essential Genes in *Rhodobacter sphaeroides*

**DOI:** 10.1128/mSystems.00015-17

**Published:** 2017-06-06

**Authors:** Brian T. Burger, Saheed Imam, Matthew J. Scarborough, Daniel R. Noguera, Timothy J. Donohue

**Affiliations:** a Great Lakes Bioenergy Research Center, Wisconsin Energy Institute, University of Wisconsin, Madison, Wisconsin, USA; b Department of Bacteriology, University of Wisconsin, Madison, Wisconsin, USA; c Department of Civil and Environmental Engineering, University of Wisconsin, Madison, Wisconsin, USA; Purdue University

**Keywords:** *Rhodobacter sphaeroides*, Tn-seq, gene disruption, genomics, metabolic modeling, metabolism, photosynthetic bacteria, proteobacteria

## Abstract

*Rhodobacter sphaeroides* is one of the best-studied alphaproteobacteria from biochemical, genetic, and genomic perspectives. To gain a better systems-level understanding of this organism, we generated a large transposon mutant library and used transposon sequencing (Tn-seq) to identify genes that are essential under several growth conditions. Using newly developed Tn-seq analysis software (TSAS), we identified 493 genes as essential for aerobic growth on a rich medium. We then used the mutant library to identify conditionally essential genes under two laboratory growth conditions, identifying 85 additional genes required for aerobic growth in a minimal medium and 31 additional genes required for photosynthetic growth. In all instances, our analyses confirmed essentiality for many known genes and identified genes not previously considered to be essential. We used the resulting Tn-seq data to refine and improve a genome-scale metabolic network model (GEM) for *R. sphaeroides*. Together, we demonstrate how genetic, genomic, and computational approaches can be combined to obtain a systems-level understanding of the genetic framework underlying metabolic diversity in bacterial species.

**IMPORTANCE** Knowledge about the role of genes under a particular growth condition is required for a holistic understanding of a bacterial cell and has implications for health, agriculture, and biotechnology. We developed the Tn-seq analysis software (TSAS) package to provide a flexible and statistically rigorous workflow for the high-throughput analysis of insertion mutant libraries, advanced the knowledge of gene essentiality in *R. sphaeroides*, and illustrated how Tn-seq data can be used to more accurately identify genes that play important roles in metabolism and other processes that are essential for cellular survival.

**Author Video**: An author video summary of this article is available.

## Abstract (precis)

Knowledge about the role of genes under a particular growth condition is required for a holistic understanding of a bacterial cell and has implications for health, agriculture, and biotechnology. We developed the Tn-seq analysis software (TSAS) package to provide a flexible and statistically rigorous workflow for the high-throughput analysis of insertion mutant libraries, advanced the knowledge of gene essentiality in *R. sphaeroides*, and illustrated how Tn-seq data can be used to more accurately identify genes that play important roles in metabolism and other processes that are essential for cellular survival.

## INTRODUCTION

*Rhodobacter sphaeroides* is a facultative purple nonsulfur phototrophic bacterium that has served as a model for the formation, function, and regulation of the photosynthetic apparatus. This metabolically diverse organism can perform aerobic and anaerobic respiration, CO_2_ fixation, N_2_ fixation, and H_2_ production and has been used as a platform in biotechnology applications (1–7). Despite its importance as a model organism, no comprehensive mutagenesis has been undertaken in *R. sphaeroides*, nor is there an extensive set of defined mutants. Our knowledge of essential genes in *R. sphaeroides* is comparative, limited to studies done in related species (8, 9), with the caveat that metabolic lifestyles and capabilities differ between closely related purple nonsulfur bacteria.

In recent years, genome-enabled experimental and computational approaches have been developed to link genes to cellular function or phenotype. Transposon sequencing (Tn-seq) is one such experimental approach, combining transposon mutagenesis with next-generation sequencing (NGS) to permit genome-wide identification of insertion sites in a library of mutants (10–13). Monitoring either the frequency of transposon insertions in a library under a single condition or changes in mutant abundance between conditions allows one to identify essential genes and the fitness contributions of nonessential genes on a genome-wide scale.

Genome-scale metabolic network reconstructions are computational frameworks, wherein genes and gene products are assigned to reactions in one or more metabolic pathways (14). The resulting genome-scale metabolic network model (GEM) can be predictive of growth, metabolite and end product accumulation, and gene essentiality. However, the predictive power of these, and other genome-scale approaches, is often limited by lack of knowledge about proteins that catalyze reactions in a pathway or the role of uncharacterized gene products under one or more conditions. Recent efforts have utilized essential gene analysis to improve the accuracy of GEMs in both eukaryotic and cyanobacterial model systems (15, 16).

In this study, we used Tn-seq to identify essential genes in *R. sphaeroides* under several growth conditions, thus establishing relationships between genes and phenotypes for bacterial genes of known and unknown function. To aid this study, we developed software that provides a streamlined and statistically rigorous workflow to identify the contributions of genes in a Tn-seq mutant library. These Tn-seq data were then used to evaluate and refine an existing genome-scale metabolic network reconstruction, with improvements to the resulting GEM. By comparing Tn-seq data to predictions of the *R. sphaeroides* metabolic model iRsp1140 (17, 18), we confirmed roles for many genes and assigned potential functions to previously uncharacterized genes. Our findings provide new insight into the lifestyles of this bacterium and illustrate how a systems-level understanding of cells can be improved by combining genome-scale experimental and computational approaches.

## RESULTS

### Workflow for Tn-seq data analysis.

**FIG 1:**
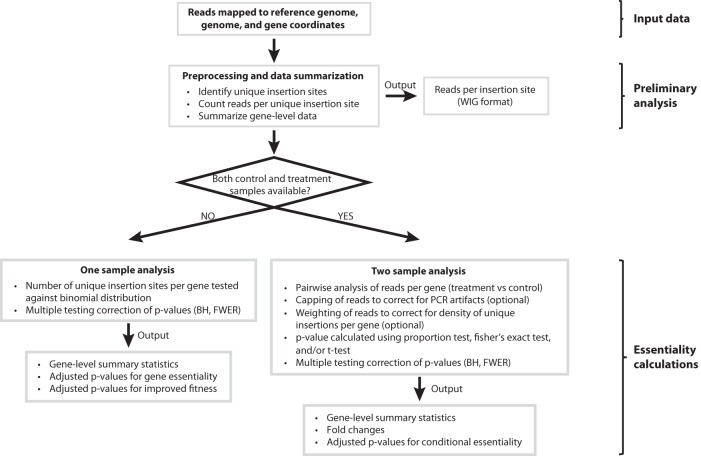
Workflow for Tn-seq analysis using TSAS. Genome sequence (FASTA format), gene coordinates (GFF v3 format), and mapped DNA sequencing reads (reads) (using Bowtie [version 1 or 2], SOAP, or ELAND) are provided as input to TSAS for essentiality analysis. After preliminary analysis to identify transposon insertion sites and count reads mapping to those sites, a one- or two-sample analysis can be performed depending on sequencing data available. See the TSAS User Guide (Text S1) for details regarding one- and two-sample analysis settings, options, and outputs. BH, Benjamini-Hochberg; FWER, family-wise error rate.

By leveraging the random insertion of transposons into a genome, Tn-seq can identify essential genes and the fitness contributions of nonessential genes on a genomic scale (10–13). This assumes that transposon insertions are often detrimental to gene function and therefore that loci that are important for growth under a given condition are less likely to harbor these genetic lesions. By analyzing the number of transposon insertions, or the frequency of DNA sequence reads mapping to these insertions, one can determine at some confidence level the likelihood that a given locus is important for growth. A number of software packages have been developed for analysis of Tn-seq data, with several of these packages designed to analyze Tn-seq data derived from mariner-based transposons, taking advantage of their thymine-adenine (TA) insertion site requirement (19–21). To improve the ability of researchers to analyze a variety of Tn-seq libraries, we developed a statistically rigorous, flexible, and streamlined software package that generates lists of important genes using NGS data of transposon libraries. Our Tn-seq analysis software (TSAS) utilizes the Java Statistical Classes application programming interface to enable rapid analysis of aligned DNA sequence reads to test the likelihood of a gene’s importance under a given condition. The TSAS workflow (Fig. 1) is organism agnostic and requires only (i) genome sequence (in FASTA format), (ii) genome coordinates (in GFF v3 format), and (iii) aligned DNA sequence reads (in Bowtie, SOAP, or Eland format).

In the first phase of the TSAS workflow, the aligned reads are used to identify and quantify transposon insertion sites across the genome. Genome coordinates are then used to determine gene-level insertion and read counts. To facilitate data visualization, the insertion location and abundance data are summarized in wiggle (WIG) format files, which can be viewed using publicly available genome browsers. Once the data for each gene have been summarized, TSAS can assess condition-dependent gene essentiality based either on a theoretical distribution of the insertions in a given sample (one-sample analysis) or on the use of a reference data set (two-sample analysis), as outlined below.

### One-sample analysis.

When using the one-sample analysis option in TSAS, the total number of insertions per gene is compared to a binomial distribution, which assumes that each base pair in the genome has an equal likelihood of a transposon insertion event. The use of a binomial distribution enables one to assess the probability of having a specific number of insertions within a locus of specified length (22). TSAS uses this probability as a proxy for the likelihood that disruption of a gene has a nonneutral impact on growth under a given condition (Fig. 1; see Text S1 [TSAS User Guide] in the supplemental material). Using a binomial distribution mitigates potential overestimation of the importance of small genes, which may have low numbers of insertions merely due to their size (see Materials and Methods). Furthermore, by using the number of unique insertions, instead of the total number of sequence reads per gene, TSAS bypasses artifacts associated with preparation of NGS libraries that can artificially inflate the frequency of DNA sequence reads at some insertion sites (23–25). The output for a one-sample TSAS analysis provides separate adjusted *P* values to assess genes having fewer insertions than expected (i.e., transposon insertions result in decreased relative fitness) or more insertions than expected (i.e., transposon insertion results in improved relative fitness) (see Text S1 [TSAS User Guide]).

10.1128/mSystems.00015-17.1 TEXT S1  TSAS user guide. This document provides detailed instructions for how to use TSAS, which provides tools for various kinds of analysis of Tn-seq data sets. Download TEXT S1, PDF file, 0.1 MB. Copyright © 2017 Burger et al. 2017 Burger et al. This content is distributed under the terms of the Creative Commons Attribution 4.0 International license.

### Two-sample analysis.

When DNA sequence data from a library grown under experimental and reference conditions are available, the user can run a two-sample TSAS analysis. In this case, the number of mapped sequence reads per gene (normalized, and with an optional bias correction) for each condition is used to calculate fold changes and associated *P* values (Fig. 1, Text S1 [TSAS User Guide], and Materials and Methods). Below, we illustrate the use of TSAS in analyzing an *R. sphaeroides* Tn-seq mutant library.

### Mutant library construction and validation.

We generated an *R. sphaeroides* Tn*5* mutant library via conjugation with a diaminopimelic acid (DAP)-auxotrophic, pRL27-containing *Escherichia coli* donor strain (WM6439). Tn*5* was chosen for this work based on the high guanine-cytosine (GC) content of *R. sphaeroides* (69% GC), which precluded the use of TA dinucleotide sequence-specific mariner-based transposons (26). Use of the WM6439 donor strain allowed for selection of kanamycin-resistant transconjugants on a rich medium (Luria-Bertani [LB]), thereby increasing the potential diversity of Tn*5* insertion sites in the master library. Colonies from master library plates were pooled, aliquoted, and stored at −80°C at ~1.2 × 10^10^ CFU/ml. As a quality assessment, we recovered transposon/genome junctions from two aliquots of this library using the Tn-seq circle method (27) and analyzed the data in a one-sample analysis with TSAS, finding ~200,000 unique insertion sites in each library aliquot (Table 1). The number of mapped DNA sequence reads per gene and insertions per gene were highly correlated between aliquots, demonstrating that we reproducibly recover transposon/genome junctions by this method (Fig. S1).

10.1128/mSystems.00015-17.2 FIG S1  Comparison of Tn*5* library aliquots. The numbers of insertions (hits) per gene (A) and reads per gene (B) were compared between two aliquots of the Tn*5* library. DNA was extracted from aliquots, and transposon/genome junctions were recovered as described in the text. Insertions per gene and reads per gene were determined by TSAS and plotted using R. Download FIG S1, TIF file, 2.1 MB. Copyright © 2017 Burger et al. 2017 Burger et al. This content is distributed under the terms of the Creative Commons Attribution 4.0 International license.

**TABLE 1:** Tn-seq analysis of *Rhodobacter sphaeroides* Tn*5* mutant library

Growth condition	No. of reads	No. (%) of mapped reads	No. of insertions^a^	Density
LB agar, aliquot 1	14,365,417	13,522,605 (94)	198,476	1/23 bp
Within ORFs		9,165,872	137,523	1/33 bp
LB agar, aliquot 2	16,845,161	15,930,130 (95)	219,688	1/21 bp
Within ORFs		10,782,481	151,908	1/30 bp
SMM,^b^ aerobic	14,830,409	13,974,109 (94)	105,310	1/44 bp
Within ORFs		9,853,418	73,659	1/62 bp
SMM,^b^ photosynthetic	13,902,604	13,427,598 (97)	111,396	1/41 bp
Within ORFs		9,404,254	77,479	1/59 bp

a Unique insertion defined as having ≥10 mapped reads.

b SMM, Sistrom’s minimal medium.

### Identifying genes required for growth on LB.

**FIG 2:**
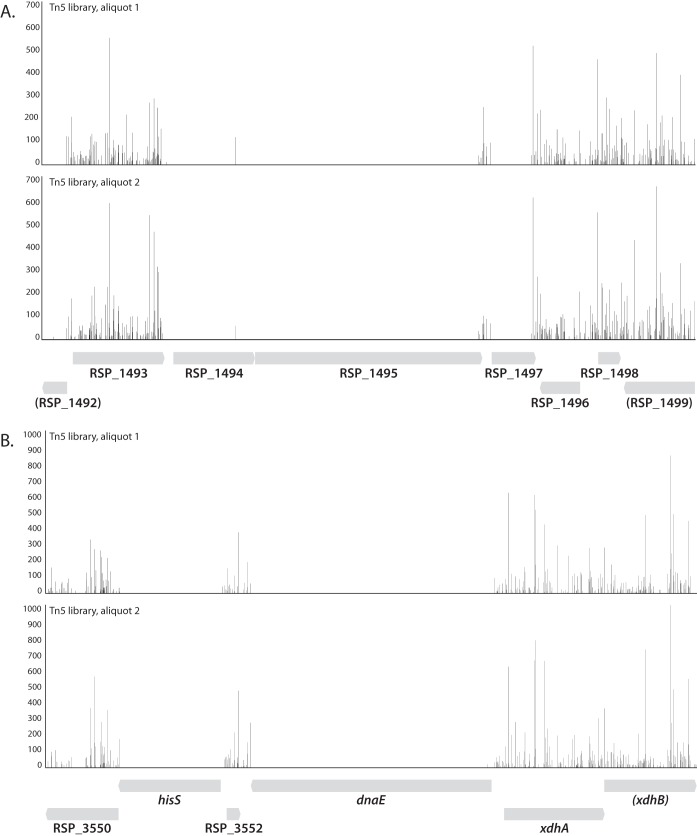
Examples of essential genes as determined by Tn-seq. An *R. sphaeroides* Tn*5* mutant library was generated on rich (LB) plates grown aerobically. Transposon insertions were determined as described in the text. (A) Genomic region surrounding the essential genes RSP_1494 (putative aspartate aminotransferase), RSP_1495 (DNA translocase FtsK), and RSP_1497 (putative outer membrane lipoprotein carrier protein). (B) Genomic region surrounding the essential genes *hisS* (RSP_3551, histidyl-tRNA synthetase) and *dnaE* (RSP_3553, DNA polymerase III, α subunit).

We performed a one-sample analysis to identify genes essential for aerobic growth on LB. We identified 839 genes with fewer insertions than expected, based on the size of the genes and the number of insertions in the library (FWER < 0.01). Of these, 493 contained fewer than four unique insertions in the central 80% of the gene (see Materials and Methods) and were classified as essential for aerobic growth on LB (Table S1). As an example, Fig. 2 shows the insertion and read count data for the genomic region surrounding several essential genes.

10.1128/mSystems.00015-17.3 TABLE S1  TSAS analysis of *R. sphaeroides* Tn*5* library. This workbook contains TSAS output for analysis of the *R. sphaeroides* Tn*5* library grown on LB. The first worksheet contains the raw TSAS output, and the second worksheet contains the essential genes, as described in the text. A description of the column headings can be found in the TSAS User Guide (Text S1). Download TABLE S1, XLSX file, 0.6 MB. Copyright © 2017 Burger et al. 2017 Burger et al. This content is distributed under the terms of the Creative Commons Attribution 4.0 International license.

**FIG 3:**
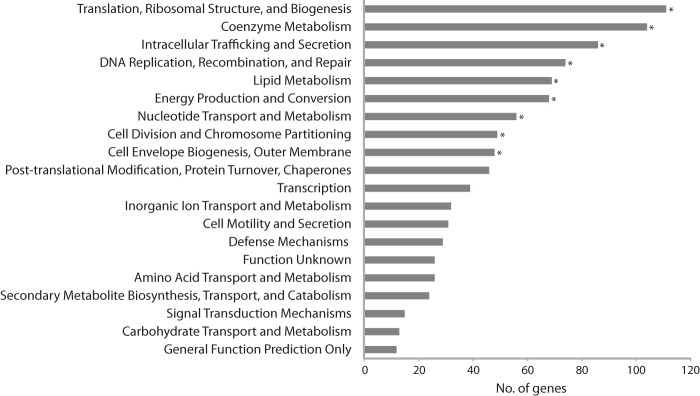
COG analysis of *R. sphaeroides* essential genes. The genes essential for aerobic growth on LB were binned by COG category. Asterisks (*) represent those categories that were overrepresented in the analysis (*P* value < 0.05, hypergeometric distribution).

We used the Clusters of Orthologous Groups of Proteins (COG) categories (28) to get an overview of the *R. sphaeroides* essential genes (Fig. 3). Nine COG categories are overrepresented in the list of essential genes (*P* value < 0.05), including many fundamental biological processes. The COG category corresponding to “Translation, Ribosomal Structure, and Biogenesis” had the greatest representation, with 111 of 271 (41%) genes present in this list.

We queried the Database of Essential Genes (DEG) (29) with the 489 protein-coding essential genes, using BLASTP to identify homologs in the bacterial database. Of these 489 genes, 431 had a putative homolog in DEG (expect threshold < 1 × 10^−10^; score > 500 [Table S2]). Thus, 88% of the genes essential for aerobic growth of *R. sphaeroides* on LB agree with gene essentiality predictions for one or more of the 39 bacteria in DEG. The 58 *R. sphaeroides* genes without a homolog in DEG, including 12 that encode hypothetical proteins, represent candidates for previously uncharacterized genes that are essential for growth on LB. For instance, in the analysis of these 58 genes, four Gene Ontology (GO) categories related to vitamin B_12_ (cobalamin) biosynthesis or transport (GO:0009236, cobalamin biosynthetic process; GO:0015420, cobalamin-transporting ATPase activity; GO:0015889, cobalamin transport; and GO:0035461, vitamin transmembrane transport) are enriched (*P* value < 0.0001), suggesting that the biosynthesis, transport, or need for cobalamin is different in *R. sphaeroides* than in the other bacteria in the DEG database, at least under the conditions tested.

10.1128/mSystems.00015-17.4 TABLE S2  Comparison of *R. sphaeroides* essential genes to DEG. This worksheet lists the *R. sphaeroides* essential genes and indicates whether a homolog for that gene is found in DEG, as determined by BLASTP (see text for details). Information regarding the organisms in DEG, including growth condition, number of essential genes, and literature reference, can be found at http://www.essentialgene.org/. Download TABLE S2, XLSX file, 0.1 MB. Copyright © 2017 Burger et al. 2017 Burger et al. This content is distributed under the terms of the Creative Commons Attribution 4.0 International license.

The properties of existing mutants can also be used as an independent test of the data derived from TSAS analysis of the transposon mutant library. For instance, RSP_1519 (*prrC*), a gene encoding a copper chaperone involved in capture and delivery of copper to cytochrome *c* oxidases (30) and also shown to have thiol-disulfide oxidoreductase activity (31), was found among the list of 489 essential genes. Deletion of *prrC* leads to increased colony pigmentation in the presence of O_2_, but loss of this gene is not reported to have a significant effect on aerobic growth on a defined medium (32). Thus, the inability to recover *prrC* mutants in the master library suggests that there is a previously unreported defect in aerobic growth on LB. The designation of *prrC* as an essential gene may also illustrate a caveat of Tn-seq, whereby slow-growing strains can erroneously be classified as essential. For example, the 14 subunits of NADH dehydrogenase (*nuoA* to -*N*) are identified by Tn-seq as essential when *R. sphaeroides* cells are grown aerobically on LB. While the presence of several of these subunits in the DEG database lends credence to their classification as essential in *R. sphaeroides*, strains lacking RSP_2521 (*nuoG*) have been observed to grow approximately twice as slow as wild-type cells under aerobic conditions in several media (33).

### Identifying genes required for growth on a minimal medium.

**FIG 4:**
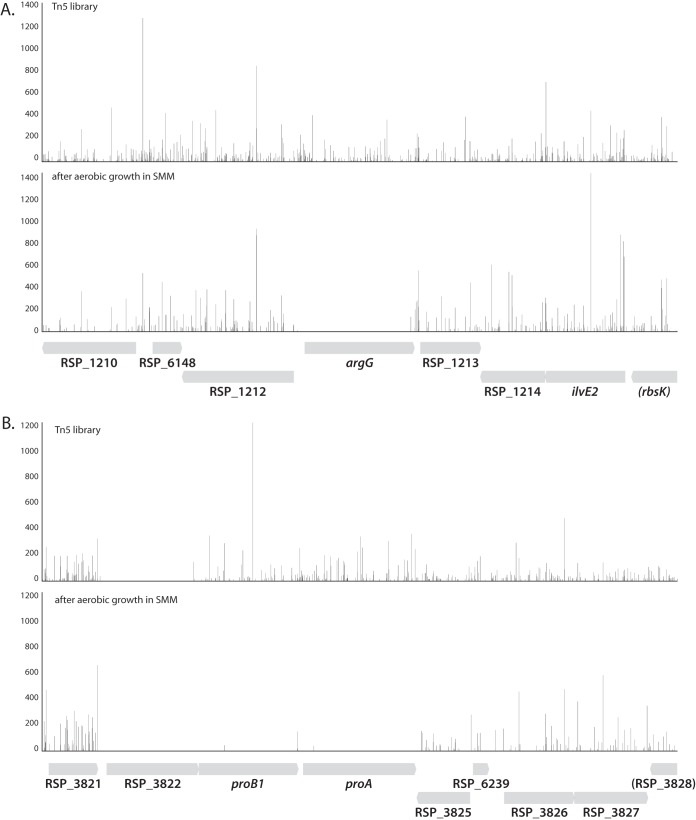
Examples of conditionally essential genes in minimal medium as determined by Tn-seq. The *R. sphaeroides* Tn*5* mutant library was outgrown aerobically in Sistrom’s minimal medium (SMM). Transposon insertions were determined as described in the text. (A) Genomic region surrounding the conditionally essential gene *argG* (RSP_1212, argininosuccinate synthase). (B) Genomic region surrounding the conditionally essential genes *proB1* (RSP_3823, glutamate 5-kinase) and *proA* (RSP_3824, glutamate-5-semialdehyde dehydrogenase). Note that RSP_3822 (GTP1/OBG family protein) was identified as essential in the Tn*5* library.

The utility of TSAS was also evaluated with a two-sample analysis to identify conditionally essential genes for aerobic growth in Sistrom’s minimal medium (SMM), which lacks exogenous amino acids and contains succinate and ammonium as the sole carbon and nitrogen sources. In this case, we grew the master library aerobically in SMM for approximately seven doublings by optical density at 600 nm (OD_600_) before preparing genomic DNA for sequencing. We identified 105 genes with fewer reads after aerobic growth in SMM compared to growth on LB (log fold change < −2; adjusted *P* value [proportions_insertions] < 0.05). Of these, 85 contained fewer than four unique insertions in the central 80% of the gene and were classified as conditionally essential for aerobic growth in SMM (Table S3; examples in Fig. 4). Since SMM lacks amino acids, we expected that many of the 85 genes identified as conditionally essential in this experiment would encode biosynthetic enzymes. Indeed, the GO terms “cellular amino acid biosynthetic process” (GO:0008652; 30 of 58 genes; *P* value = 8.02 × 10^−41^), “purine nucleotide biosynthetic process” (GO:0006164; 10 of 14 genes; *P* value = 2.7 × 10^−17^), and “pyrimidine nucleotide biosynthetic process” (GO:0006221; 6 of 10 genes; *P* value = 9.4 × 10^−11^) are overrepresented in 46 of these 85 genes. In addition, most of the other 39 genes that are identified as essential for aerobic growth in SMM are annotated as encoding biosynthetic functions.

10.1128/mSystems.00015-17.5 TABLE S3  TSAS analysis of *R. sphaeroides* Tn*5* library grown aerobically in SMM. This workbook contains TSAS output for analysis of the *R. sphaeroides* Tn*5* library after aerobic growth in SMM. The first worksheet contains the raw TSAS output, and the second worksheet contains the conditionally essential genes, as described in the text. A description of the column headings can be found in the TSAS User Guide (Text S1). Download TABLE S3, XLSX file, 1.2 MB. Copyright © 2017 Burger et al. 2017 Burger et al. This content is distributed under the terms of the Creative Commons Attribution 4.0 International license.

**FIG 5:**
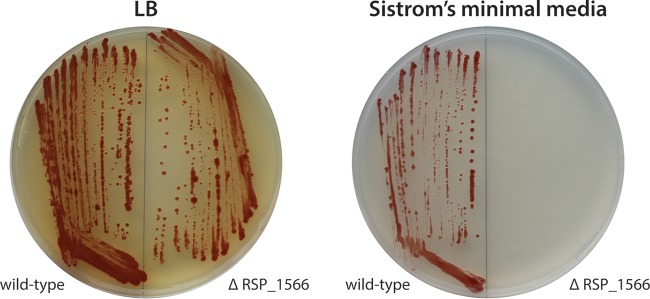
Confirmation of essential gene identified by Tn-seq analysis. *R. sphaeroides* 2.4.1 wild-type and ΔRSP_1566 were streaked to LB and Sistrom’s minimal medium (SMM) agar plates and incubated at 30°C for 4 days. Wild-type cells grow on both LB and SMM plates, but ΔRSP_1566 grows only on LB, as predicted by Tn-seq analysis.

Included in the remaining genes identified as conditionally essential for aerobic growth in SMM are four that encode hypothetical proteins. When we generated a markerless, in-frame deletion mutant for one of these genes (RSP_1566), we found that this strain was able to grow aerobically on LB plates but was unable to grow aerobically on SMM plates (Fig. 5), demonstrating that the Tn-seq analysis accurately identified conditional essentiality for this gene. This gene product is predicted to contain a CbiX/SirB chelatase domain that often functions in siroheme or vitamin B_12_ synthesis (34), suggesting that RSP_1566 plays a previously unknown role in inserting metal into sirohydrochlorin under aerobic conditions. Another hypothetical protein identified as essential for aerobic growth in SMM, RSP_6139, is likely to be involved in amino acid biosynthesis since it is potentially cotranscribed with genes encoding the two subunits of the isopropylmalate isomerase (*leuD* and *leuC*) in the leucine biosynthetic pathway. Two other genes identified as essential for aerobic growth in SMM, RSP_1147 and RSP_1148, are potentially cotranscribed with neighboring genes encoding the large and small subunits of glutamate synthase (*gltB* and *gltD*, respectively), suggesting that these hypothetical proteins also play a previously unrealized role in amino acid biosynthesis.

### Identifying genes required for photosynthetic growth.

**FIG 6:**
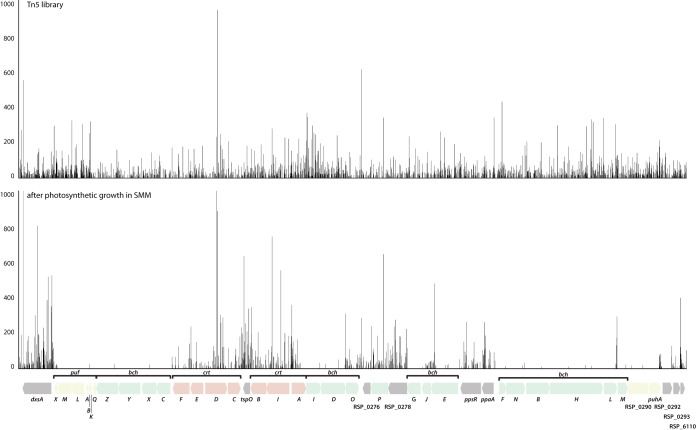
Negative selection in the photosynthetic gene cluster of *R. sphaeroides* after photosynthetic growth in SMM. The *R. sphaeroides* Tn*5* mutant library was outgrown photosynthetically in Sistrom’s minimal medium (SMM). Transposon insertions were determined as described in the text. The genomic region encompassing the photosynthetic gene cluster is shown, before and after photosynthetic growth in SMM. Genes are colored according to their annotation (*puf*, yellow; *bch*, green; *crt*, pink).

To identify genes required for photosynthetic growth, we grew the library photosynthetically for seven doublings by OD_600_ in SMM before determining which genes had undergone selection. In a two-sample analysis (using aerobic growth on LB as a control), we identified 146 genes with fewer reads after photosynthetic growth in SMM (log fold change < −2; adjusted *P* value [proportions_insertions] < 0.05). Of these, 91 had fewer than four insertions in the central 80% of the gene and are considered conditionally essential for photosynthetic growth in SMM. In order to remove the effect of growth in a minimal medium, we excluded genes essential for aerobic growth in SMM (see above), leaving 31 genes required for photosynthetic growth (Table S4). Of these 31 genes, 14 are found in the so-called photosynthetic gene cluster (PGC), a group of genes known to encode components of the photosynthetic apparatus in this and related purple nonsulfur bacteria (35) (Fig. 6). The transcriptional regulator CbbR, responsible for regulating genes of the reductive pentose phosphate pathway (Calvin cycle) (36–38), was also classified as conditionally essential for photosynthetic growth. Several genes not previously identified as having a role in photosynthesis, such as RSP_2885 (*glgA*; glycogen synthase), RSP_0465 (putative protease), and RSP_2947 (glutamate racemase), were identified as conditionally essential in this analysis. In addition, four genes encoding hypothetical proteins were also classified as essential for photosynthetic growth (Table S4). Future experiments are needed to test the role and function of these genes in photosynthetic growth.

10.1128/mSystems.00015-17.6 TABLE S4  TSAS analysis of *R. sphaeroides* Tn*5* library grown photosynthetically in SMM. This workbook contains TSAS output for analysis of the *R. sphaeroides* Tn*5* library after photosynthetic growth in SMM. The first worksheet contains the raw TSAS output, and the second worksheet contains the genes conditionally essential for photosynthetic growth in SMM. The third worksheet contains those genes essential for photosynthetic growth after removing genes essential for aerobic growth in SMM (see text for details). A description of the column headings can be found in the TSAS User Guide (Text S1). Download TABLE S4, XLSX file, 1.2 MB. Copyright © 2017 Burger et al. 2017 Burger et al. This content is distributed under the terms of the Creative Commons Attribution 4.0 International license.

### Using Tn-seq data to refine a metabolic model.

iRsp1140 is a GEM for *R. sphaeroides* 2.4.1 consisting of 878 metabolites, 1,416 reactions, and 1,137 genes, covering about 25% of the predicted open reading frames (18, 39). Essential genes can be predicted with iRsp1140 by systematic *in silico* deletion of individual genes followed by assessment of whether the model predicts growth (40, 41). To compare essential genes identified with Tn-seq to those predicted by iRsp1140, we performed separate one-sample analyses of the Tn*5* library grown aerobically in SMM and photosynthetically in SMM.

**FIG 7:**
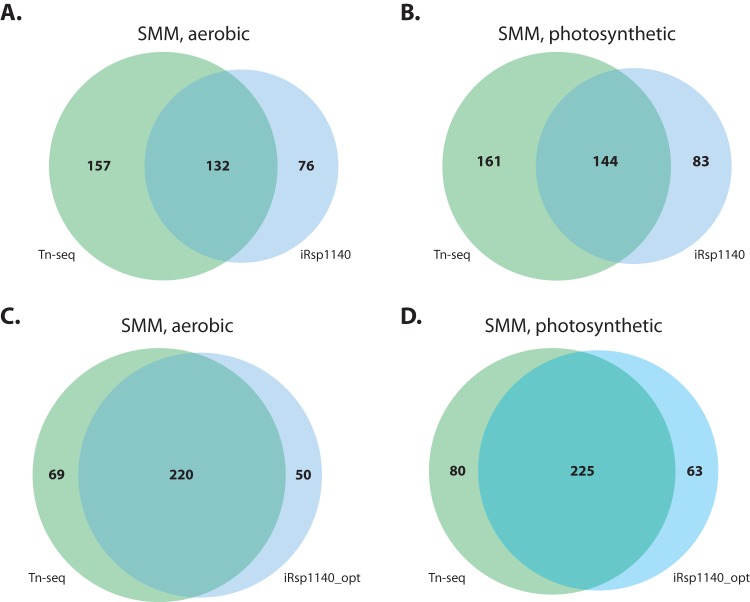
Comparing TSAS- and iRsp1140-predicted essential genes. We used TSAS to identify genes essential for aerobic and photosynthetic growth in Sistrom’s minimal medium and compared these to iRsp1140 essential gene predictions (aerobic [A] and photosynthetic [B]). We then updated iRsp1140 based on inspection of the genes that were predicted to be essential by either Tn-seq or TSAS alone, resulting in iRsp1140_opt. These changes to the model resulted in increased agreement with TSAS-predicted essential genes for aerobic (C) and photosynthetic (D) growth.

A one-sample analysis of the library grown aerobically in SMM identified 501 essential genes (FWER < 0.01; <4 insertions in the central 80% of the gene), of which 289 are included in iRsp1140 (Table S5). In parallel, *in silico* simulations with iRsp1140 predicted 208 single-gene deletions that result in no aerobic growth in SMM. A comparison of the two data sets (Fig. 7A) shows that the TSAS and iRsp1140 analyses overlap in 132 essential genes (*P* value = 2.9 × 10^−40^, hypergeometric test). When we used a similar scheme to compare Tn-seq data and iRsp1140 essential gene predictions for photosynthetic growth in SMM (Fig. 7B), we again found significant but incomplete overlap (144 genes; *P* value = 6.6 × 10^−41^, hypergeometric test). Because the libraries were grown in SMM, there is considerable overlap between aerobic and photosynthetic analyses when considering TSAS-specific genes, iRsp1140-specific genes, and the intersect of essentiality predicted by TSAS and iRsp1140. For example, 64 of the 83 genes predicted to be essential for photosynthetic growth in SMM by iRsp1140 alone were also included in the list of iRsp1140-specific essential genes for aerobic growth in SMM.

10.1128/mSystems.00015-17.7 TABLE S5  Comparison of TSAS- and iRsp1140-predicted essential genes. This workbook includes six worksheets: raw TSAS output for a one-sample analysis of the *R. sphaeroides* Tn*5* library grown aerobically in SMM, raw TSAS output for a one-sample analysis of the *R. sphaeroides* Tn*5* library grown photosynthetically in SMM, results of a Venn diagram analysis of TSAS- and iRsp1140-predicted essential genes for aerobic growth in SMM, results of a Venn diagram analysis of TSAS- and iRsp1140-predicted essential genes for photosynthetic growth in SMM, results of a Venn diagram analysis of TSAS- and iRsp1140_opt-predicted essential genes for aerobic growth in SMM, and results of a Venn diagram analysis of TSAS- and iRsp1140_opt-predicted essential genes for photosynthetic growth in SMM. Download TABLE S5, XLSX file, 1.1 MB. Copyright © 2017 Burger et al. 2017 Burger et al. This content is distributed under the terms of the Creative Commons Attribution 4.0 International license.

The genes called essential either by TSAS or by iRsp1140 alone could provide insight into modifications that would improve iRsp1140’s predictive ability. For instance, if a gene is identified as essential by TSAS but not iRsp1140, and the reaction catalyzed by the gene is known to be essential, then the set of isozymes assigned to the reaction in the metabolic model may need to be reduced. This was the case with RSP_2172 {*metF*, 5,10-methylenetetrahydrofolate reductase [NAD(P)]} and RSP_2769 (putative 5,10-methylenetetrahydrofolate reductase), both associated in iRsp1140 with an essential reaction for tetrahydrofolate biosynthesis. Because only RSP_2172 was identified as essential by TSAS, the gene-protein-reaction (GPR) rule for this reaction was modified to include only RSP_2172, thus making the essentiality for this reaction agree between TSAS and iRsp1140. For other reactions catalyzed by genes called essential by TSAS that were not essential in iRsp1140, we investigated if whole pathways should be added to iRsp1140 or made essential through the addition of the products of these reactions to the biomass equation. This resulted in the addition of vitamin B_6_ and vitamin B_12_ to the biomass equation for growth in SMM, with whole pathways added for anaerobic synthesis of vitamins B_6_ via 5-amino-1-(5-phospo-d-ribosyl)imidazole (42) and vitamin B_12_ via hydroxypyruvate (43–46).

Another approach to increase compatibility between TSAS and iRsp1140 predictions was to reevaluate reaction reversibility in selected pathways. For instance, iRsp1140 allowed the use of amino acid degradation pathways for both turnover and *de novo* synthesis of amino acids, thus resulting in predictions of nonessentiality for genes in their biosynthetic pathways. This was corrected using BioCyc (43, 44) to guide modification of reaction reversibility, where necessary. In another example, iRsp1140 allowed for aerobic production of gluconeogenesis intermediates using the anaerobic Calvin cycle, resulting in many gluconeogenesis reactions being predicted as nonessential. To correct this, we modified model constraints, turning off known anaerobic growth-specific pathways by setting flux through these pathways to zero when growing aerobically. Also, when growing photosynthetically (anaerobically in the light), iRsp1140 could use oxygen for synthesis of some essential biomass compounds. We again modified model constraints, turning off pathways for oxygen-dependent porphyrin synthesis and vitamin B_6_ synthesis during anaerobic growth simulations.

We also examined genes called essential by iRsp1140 but not by TSAS. In these cases, modifying GPR rules for essential reactions was difficult as there were often no known alternative enzymes capable of performing this function. We did, however, modify GPRs for several of the tRNA synthesis genes to match the essentiality calls from TSAS analysis. Another change based on the Tn-seq data was to remove the carotenoid spheroidenone from the aerobic biomass equation. This change alone supported removal of six of the iRsp1140-only genes, as the genes encoding enzymes responsible for spheroidenone biosynthesis are no longer considered essential.

In total, by integrating the Tn-seq data with iRsp1140, 10 reactions were deleted, while 21 reactions, 12 metabolites, and 5 genes were added. Reaction reversibility was modified for 17 reactions, and GPRs were modified for 87 reactions (see Table S6 for a complete list of changes). These changes resulted in a new instance of iRsp1140, called iRsp1140_opt, which showed increased overlap with genes that are identified as essential by Tn-seq (Fig. 7). For aerobic conditions, the number of overlapping genes increased from 132 (36%) to 220 (65%) (*P* value = 3.7 × 10^−123^, hypergeometric test), while the number of overlapping genes increased from 144 (37%) to 225 (61%) (*P* value = 3.2 × 10^−110^, hypergeometric test) for photosynthetic conditions. In sum, these refinements highlight the utility of comparing genome-scale data sets to metabolic network reconstructions as a way to improve metabolic network models.

10.1128/mSystems.00015-17.8 TABLE S6  Modifications to iRsp1140. This workbook contains details about changes made to iRsp1140 based on gene essentiality determined with Tn-seq. The workbook includes four worksheets: genes predicted to be essential under aerobic conditions with Tn-seq but not predicted to be essential with iRsp1140, genes predicted to be essential under aerobic conditions with iRsp1140 but not with Tn-seq, genes predicted to be essential under photosynthetic conditions with Tn-seq but not predicted to be essential with iRsp1140, and genes predicted to be essential under photosynthetic conditions with iRsp1140 but not with Tn-seq. Each worksheet includes the gene locus tag; the annotation for the protein product; reactions in the model for which the gene is involved; the overall gene-protein-reaction rule including the gene; comments related to the involved pathway and whether a change to the model is necessary; the implemented model change (if any); and, if the change was made, whether the gene essentiality changed. The tabs for photosynthetic conditions also include whether or not the gene was essential under aerobic conditions. Download TABLE S6, XLSX file, 0.1 MB. Copyright © 2017 Burger et al. 2017 Burger et al. This content is distributed under the terms of the Creative Commons Attribution 4.0 International license.

## DISCUSSION

In this study, we show how genetic, genomic, and computational approaches can be used to increase our knowledge of the roles of individual genes under different growth conditions. New advances and insights derived from this work are discussed below.

### Software for analyzing Tn-seq data.

In recent years, Tn-seq studies have identified essential genes in a wide variety of bacteria, using different experimental and computational approaches (10–13, 27, 47–49). Here, we introduced a new Tn-seq data analysis package, TSAS, which is approachable (requiring no additional programs [aside from Bowtie] and minimal command line interaction) and flexible (able to handle both one- and two-sample analyses) and provides the users with tools to perform a statistically rigorous analysis of their data. In this paper, we have highlighted the use of TSAS to identify essential and conditionally essential genes using one- and two-sample analyses, respectively. While not explored in-depth here, TSAS is also capable of uncovering genes with positive or negative fitness contributions. Aside from those genes considered essential, genes with fewer insertions than expected (one-sample analysis) or that undergo negative selection after outgrowth (fewer reads, two-sample analysis) represent genes that contribute in a positive manner to the fitness of the organism. Conversely, genes with more insertions than expected (one-sample analysis) or that undergo positive selection after outgrowth (more reads, two-sample analysis) represent those that contribute in a negative manner to the fitness of the organism. Researchers may find either set of genes useful during strain design (for a particular phenotype) or when insertion of foreign DNA into a nonnecessary part of the genome is required (insertion of a synthetic operon, for example).

### Identifying essential genes with TSAS.

We used TSAS to identify, for the first time, genes that are essential for aerobic growth of *R. sphaeroides* on LB. With 429 of the 489 protein-encoding genes identified as essential in *R. sphaeroides* having a homolog in the DEG database, we propose that there is high confidence in the lists of essential *R. sphaeroides* genes. When we compared the 489 genes to 522 genes of *Rhodopseudomonas palustris* (a closely related purple nonsulfur bacterium) recently reported to be essential for growth in rich medium (9), there were 322 in common. In a comparison of the 489 *R. sphaeroides* genes identified as essential in a rich medium with the 480 essential genes in the alphaproteobacterium *Caulobacter crescentus* (50), there is an overlap of 326 genes. As expected, the majority of the core essential genes in these three species encode proteins involved in conserved housekeeping functions like cell division, DNA replication, transcription, and translation.

We also applied TSAS to the *R. sphaeroides* Tn*5* library grown aerobically in SMM to test its ability to identify genes that are required for growth in a minimal medium. Not surprisingly, our analysis revealed that many of the conditionally essential genes for aerobic growth in SMM are known or predicted to synthesize the amino acids, purines, pyrimidines, and other cellular constituents that are absent in this medium (Table S3). We also showed that deletion of RSP_1566, one of the four previously uncharacterized hypothetical proteins identified as essential for aerobic growth in SMM, was lethal under these conditions (Fig. 5). We are currently testing the role of other hypothetical proteins in aerobic conditions in SMM.

We also tested the utility of this approach in identifying the role of genes in photosynthetic growth. As expected, many of the genes identified by TSAS to be conditionally essential for photosynthetic growth mapped to the PGC (Fig. 6), a genomic region containing photosynthesis-related genes in *R. sphaeroides* and related bacteria (51–54). These essential genes include many of the bacteriochlorophyll biosynthesis (*bch*) genes and genes encoding pigment-binding proteins of the photosynthetic apparatus (*puhA* and *pufLM*, encoding reaction center subunits; *pufBA*, encoding subunits of the B870 light-harvesting I complex; and RSP_0290, encoding a light-harvesting I [B870] complex assembly protein) that have been shown previously to be required for photosynthetic growth (52).

Our analysis supports previous conclusions that not all genes in the PGC are required for photosynthetic growth. For instance, a previous study using localized mutagenesis of the *R. sphaeroides* PGC recovered photosynthetically competent strains containing insertions in all of the carotenoid biosynthesis (*crt*) genes with the exception of *crtF* (52). It was not surprising that our Tn-seq data showed that none of the *crt* genes were essential since carotenoids are accessory pigments not directly involved in light energy capture (55). The gene encoding the PufX protein (RSP_0255) in the PGC has been shown to be required for photosynthetic growth (56, 57), though photosynthetic growth can restored in *pufX* mutants with second-site mutations in the light-harvesting complex B875 (58, 59). Our data classified *pufX* as nonessential, and while such discrepancies may be explained by secondary mutations, differences in light intensities or methods used to score photosynthetic growth, or statistical cutoffs used for TSAS analysis, they illustrate the need to confirm the findings made by analyzing Tn-seq libraries. In this regard, our analysis predicts a role for several newly identified or unexpected genes in photosynthetic growth, including four hypothetical genes and others encoding homologs of glycogen synthase and glutamate racemase enzymes. Future analysis of these genes could identify new functions that are important for the photosynthetic lifestyle of *R. sphaeroides* and related photosynthetic bacteria.

### Comparison of essential genes to those predicted by GEMs.

The ability of TSAS to correctly identify genes that are essential for growth motivated us to compare these genes to those predicted by the *R. sphaeroides* GEM. When we compared the essential genes identified by TSAS with those predicted by iRsp1140, we found a high degree of agreement between the two data sets, which was improved by refining the metabolic model using the Tn-seq data (Fig. 7). We identified genes called essential by iRsp1140 or TSAS alone and made targeted changes to iRsp1140 to reconcile these differences. By modifying GPR rules and biomass composition, adding or deleting reactions, modifying reaction reversibility, or turning off certain reactions in iRsp1140 under defined growth conditions, we were able to achieve better agreement between the metabolic model and the Tn-seq data sets. To our knowledge, this is one of the few comparisons between essential gene predictions by flux balance analysis (FBA) and analysis of a large-scale transposon mutant library (15, 16).

Of the remaining genes for which TSAS and iRsp1140 disagree, many of these differences likely reflect the limitations of metabolic models. For instance, several tricarboxylic acid (TCA) cycle genes are identified as essential by TSAS, but iRsp1140 can cycle metabolites through amino acid and nucleotide synthesis pathways to produce the needed intermediates. However, iRsp1140 is likely making erroneous predictions since flux through nucleotide and amino acid synthesis reactions would be low compared to that through central carbon metabolism. While iRsp1140 predicts lower growth rates when these nonessential TCA cycle genes are deleted, the reduction is only 2 to 5% lower than the optimal growth rate. By further constraining maximum fluxes through nucleotide and amino acid synthesis, iRsp1140 would predict lower growth rates. In addition, ATP synthase genes are considered nonessential in iRsp1140 but are identified as essential by TSAS. While growth in iRsp1140 is only about 10% of optimum when ATP synthase genes are deleted, the model predicts that cells acquire enough energy through substrate-level phosphorylation to maintain growth, albeit at a reduced rate.

For some genes identified as essential with TSAS but not with iRsp1140, it is likely that they have additional functions or broad metabolic functions. One example is a SpoT/ RelA family protein (RSP_1670) that is associated with a nonessential reaction in iRsp1140. It is expected, however, that this gene plays an essential role in transcriptional regulation (60, 61). Furthermore, some genes, such as RSP_2779 (*catA*, catalase), are involved in quenching reactive oxygen species. In the future, accounting for stress response and regulatory mechanisms to achieve more holistic biological models should further increase the agreement of gene essentiality predictions with Tn-seq data.

Additionally, TSAS shows that *R. sphaeroides* may have preferred pathways for synthesizing essential biomass components such as nucleotides. While known alternative nucleotide synthesis pathways exist, and *R. sphaeroides* contains some genes for these alternative pathways, Tn-seq data show that specific pathways are dominant. It should be noted that *R. sphaeroides* may be able to utilize these alternate pathways at a sufficient capacity to support growth under specific conditions.

Comparisons between TSAS and iRsp1140 provided some feedback on the statistical cutoffs used in the TSAS analyses, as well as highlighting the limitations of a statistically rigorous approach to calling essentiality. For example, there were 76 genes predicted only by iRsp1140 to be essential for aerobic growth in SMM. Of these, 37 genes contained fewer than four transposon insertions and yet were not called essential in our TSAS analysis. Eleven of these 37 genes would have been called essential with a less-stringent adjusted *P* value cutoff in TSAS, but the remaining 26 genes provide some insight into the limitations of one-sample analyses in reliably calling genes of small size as essential. In these cases, the small size of the genes and the number of insertions in the library prevent them being called essential, regardless of the statistical cutoff.

In sum, we have shown that the knowledge acquired by statistical analysis of Tn-seq libraries can have many uses. Analysis of genome-scale mutant libraries, when coupled with metabolic modeling, high-throughput phenotyping, and other studies that probe gene-function relationships, should provide a way to identify important growth capabilities and the essential underlying genes.

## MATERIALS AND METHODS

### Bacterial strains and growth conditions.

*R. sphaeroides* 2.4.1 was used in this study. WM6439 is an auxotrophic *E. coli* strain requiring 2,6-diaminopimelic acid (DAP) for growth. *R. sphaeroides* was grown at 30°C in SMM (62) and supplemented with 25 μg/ml kanamycin, when required. WM6439 was grown at 37°C in LB supplemented with 50 μg/ml kanamycin and 0.1 mM DAP.

### Transposon library construction.

*R. sphaeroides* was mutagenized by Tn*5* after conjugation with *E. coli* WM6439 containing plasmid pRL27 (63). Briefly, *E. coli* WM6439(pRL27) and *R. sphaeroides* 2.4.1 cells in mid-exponential phase were mixed 1:1, pelleted, resuspended in LB supplemented with 0.1 mM DAP, spotted onto LB plates containing 0.1 mM DAP, and incubated for 3 h at 30°C. Cells were recovered from plates with liquid LB, pelleted, resuspended in LB with glycerol (25% [vol/vol] final concentration), frozen in a dry ice-ethanol bath, and stored at −80°C. The mixtures from two independent matings were thawed, plated on LB agar supplemented with 25 μg/ml kanamycin, and incubated at 30°C for 7 days. Kanamycin-resistant *R. sphaeroides* colonies were scraped from the plates, resuspended in LB supplemented with 25 μg/ml kanamycin, mixed with glycerol to a final concentration of 25% (vol/vol), aliquoted, frozen in a dry ice-ethanol bath, and stored at −80°C. The final concentration of cells in the mutant library is 1.2 × 10^10^ CFU/ml.

### Transposon library propagation.

For outgrowth experiments, 50 μl of the frozen library was used to inoculate cultures containing 500 ml SMM supplemented with 25 μg/ml kanamycin. Aerobic cultures were sparged with 69% N_2_, 30% O_2_, and 1% CO_2_. Photosynthetic cultures were sparged with 99% N_2_ and 1% CO_2_. Cultures were incubated for approximately seven doublings, as monitored by OD_600_. Cells were collected by centrifugation for 10 min at 5,000 × *g*, washed with 25 ml phosphate-buffered saline (PBS), collected by centrifugation for 5 min at 5000 × *g*, resuspended in 2 ml PBS, mixed with glycerol to a final concentration of 25% (vol/vol), frozen, and stored at −80°C.

### Tn-seq sample preparation.

Transposon-genome junctions were recovered using a modified version of the Tn-seq circle method (27). Briefly, genomic DNA was isolated using a DNeasy Blood and Tissue kit (Qiagen), including the RNase A digestion step, and sheared to an average size of 400 bp using a Misonix S4000 sonicator with cup horn 431C and the following settings: 50% amplitude and 6 periods of 30 s on/off. Sheared DNA was purified and concentrated using a QIAquick PCR purification kit (Qiagen). Five micrograms of sheared DNA for each sample was used as input for the following enzymatic steps. End repair, A-tailing, and adapter ligation were performed using the Kapa DNA library preparation kit for Illumina (Kapa Biosystems). Illumina-compatible NEXTflex PCR-free barcode adapters were purchased from Bioo Scientific. Enzymatic cleanups between steps were performed with Agencourt AMPure XP magnetic beads (Beckman Coulter, Inc.). Adapter-ligated DNA was digested with HindIII-HF (New England Biolabs) and dual size selected (0.6× and 0.8× cuts) using AMPure XP magnetic beads (Beckman Coulter, Inc.). Circularization and exonuclease treatment followed previously described procedures (27), using Collector_oligo. DNA was amplified with Kapa Hi-Fi polymerase (Kapa Biosystems) according to the manufacturer’s protocol for 24 to 26 cycles (SLXA_FOR_AMP2 and index-appropriate REV_AMP primers). DNA was quantified using an Illumina ABI Prism library quantification kit Kapa Biosystems) on a 7500 real-time PCR system (Applied Biosystems). Libraries were sequenced on an Illumina HiSeq2000 sequencer (100 bp, single read) at the University of Wisconsin Biotechnology Center using Tn-seq_Illumina_primer. See Table S7 in the supplemental material for primer sequences.

10.1128/mSystems.00015-17.9 TABLE S7  Primer sequences. Download TABLE S7, XLSX file, 0.02 MB. Copyright © 2017 Burger et al. 2017 Burger et al. This content is distributed under the terms of the Creative Commons Attribution 4.0 International license.

### Tn-seq data analysis.

Illumina sequencing data were mapped to the *R. sphaeroides* 2.4.1 genome (RefSeq assembly accession no. GCF_000012905.2, Assembly:ASM1290v2) using Bowtie version 1.0.0 (64) with default parameters, except that 50 bases were trimmed from the low-quality (right) end of each read before alignment and only one read was reported (at random) for reads with more than one reportable alignment.

TSAS requires organism-specific data files to process data from a Tn-seq experiment. The following three files are required to run TSAS: (i) aligned read file (i.e., output from short-read alignment software, which maps reads from sequencing experiments to the reference genome; Bowtie, SOAP or Eland result formatted files are accepted), (ii) genome sequence of the target organism in FASTA format, and (iii) genomic coordinates in GFF v3 format. These files are used to assign reads and insertion sites to the genes in which they occur and provide the basis for downstream statistical analysis.

Some common issues with data collected for Tn-seq analysis include bias from library construction, PCR amplification, and nonrandom transposon insertion. To correct for some of these biases, TSAS provides two options. The first is read capping. PCR amplification during sample preparation could exaggerate the number of reads at specific locations. Similarly, hot spots for transposon insertion (if any) could also result in inflated read counts. To correct for these potential biases in the data, we implement a read capping routine that places an upper bound on the allowable number of reads permitted at a given insertion site. Three options are provided to the user: (i) mean of the reads per unique insertion across the genome plus 2 standard deviations, (ii) mean of the reads per unique insertion across the genome, and (iii) median of the reads per unique insertion across the genome. The first option in read capping is implemented by default. The second TSAS option is read weighting: the use of NGS for Tn-seq analysis means that a wealth of DNA sequencing reads is generated for each experiment. While the observed number of DNA sequencing reads per gene is likely anticorrelated with its importance under a given condition, we postulate that the number of insertions per gene is a more important metric for assessing essentiality. Thus, to emphasize the importance of insertions in gene essentiality estimations, while still leveraging the abundance of read count data obtained from NGS, we implemented an additional correction routine that gives greater weight due to the presence of larger numbers of unique insertions: total no. of reads (*i*) × normalized unique insertions (*i*)/average normalized unique insertions per gene, where total no. of reads (*i*) is the total number of reads for gene *i*, normalized unique insertions (*i*) is the total number of unique insertion for gene *i* divided by the length of gene *i*, and average normalized unique insertions per gene is defined as

$$\frac{1}{n}\sum_{i=1}^{n}\text{normalized unique insertions }\left(i\right)$$

where *n* is the total number of genes in the genome. The correction essentially results in an increase in number of reads per gene for genes whose number of normalized insertions is greater than the average and vice versa, weighting the observed reads based on insertions.

In addition to enabling “read capping” and “read weighting,” TSAS provides the user an option of setting thresholds on the number of reads mapping to a given insertion site required for that insertion site to be considered for downstream analysis (min_hits), as well as the percentage of the 5′ and 3′ ends of the genes that may be omitted during statistical analysis as they may not contribute to gene function. For the analyses presented here, 10% of the 5′ and 3′ ends of genes were excluded from analysis and the minimum hit threshold was set to 10 mapped reads. We allowed four insertions per gene in our essential genes, which provided a buffer against insertions that fell inside the 10% excluded from the 5′ and 3′ ends.

To make calls on gene essentiality, the summarized gene-level read and insertion counts are statistically evaluated in TSAS using either a one- or a two-sample analysis.

### (i) One-sample analysis.

When only one condition is available for analysis, a one-sample analysis is run. For this analysis, the observed number of unique insertions is compared to a binomial distribution to determine the probability (*P*) that a gene is essential:

$$P\left(k_{i},n_{i}\right)=\left(\begin{array}{c} n_{i}\\ k_{i} \end{array}\right)p^{k_{i}}\cdot {\left(1-p\right)}^{n_{i}\;-\;k_{i}}$$

where *n*_*i*_ is the length of gene *i* in base pairs, *k*_*i*_ is the number of observed unique insertions in gene *i*, and *p* is the probability of insertion across the entire genome (i.e., total number of unique insertions divided by genome size in base pairs). Only the lower tail probability (i.e., *P*[*X* ≤ *x*]) is calculated to obtain a *P* value for essentiality. The *P* value for improved fitness (i.e., *P*[*X* ≥ *x*] or 1 − *P*[*X* ≤ *x*]) is determined to identify genes that may be disadvantageous for growth under the condition of interest. The current implementation assumes a random insertion of the transposon across the genome with no sequence preference and thus may not be suitable for Tn-seq data from site-specific transposons, for which gene-specific probabilities also have to be considered. Use of the binomial distribution helps reduce false-positive essentiality calls for small genes. For instance, if the insertion density of the library is 1 every 500 bp, a 100-bp gene will have a very low probability of getting an insertion. Thus, if the absence of intragenic insertions was used as the criterion for essentiality, such small genes would be deemed essential purely by chance.

### (ii) Two-sample analysis.

When both a reference and a treatment condition are available, the two-sample analysis approach can be employed. For this analysis, the fold change in the total number of reads per gene is calculated as:

$${\text{FC}}_{i}=\frac{{\text{total reads treatment}}_{\left(i,\text{cap},\text{weight}\right)}/\overset{n}{\underset{i=1}{\sum }}{\text{total reads treatment}}_{\left(i,\text{cap},\text{weight}\right)}}{{\text{total reads reference}}_{\left(i,\text{cap},\text{weight}\right)}/\overset{n}{\underset{i=1}{\sum }}{\text{total reads reference}}_{\left(i,\text{cap},\text{weight}\right)}}$$

where FC_*i*_ is the fold change for gene *i* in the treatment condition relative to the reference, total reads treatment_(*i*,cap,weight)_ is the total number of reads counted for gene *i* (which may be optionally capped and weighted), and total reads reference_(*i*,cap,weight)_ is the total number of reads counted for gene *i* (which may be optionally capped and weighted). Normalization of the bias-corrected reads is achieved through division by the total number of reads for each data set. To determine the statistical significance of the calculated fold changes, *P* values are calculated using (i) Fisher’s exact test, (ii) a proportions-based test, and (iii) Student’s *t* test. The third option is implemented only when a sufficient number of replicate samples is provided.

The *P* values generated from the one- and two-sample analyses are corrected for multiple testing to generate either a family-wise error rate (Bonferroni correction [65–67]) or an adjusted *P* value (Benjamini-Hochberg correction [68]).

All source code required to run TSAS can be obtained from GitHub at https://github.com/srimam/TSAS. A detailed description of how to use TSAS and interpret the results generated is provided in the TSAS User Guide (Text S1).

### Mutant construction.

An in-frame, markerless deletion of RSP_1566 was constructed using the suicide vector pK18*mobsacB* (69). Briefly, RSP_1566 plus ~1-kbp flanking DNA up- and downstream of RSP_1566 was amplified from *R. sphaeroides* genomic DNA with primers g1566_F_XbaI and g1566_R_HindIII. The amplified products were digested with XbaI and HindIII restriction enzymes (New England Biolabs) and ligated into pK18*mobsacB* to generate pK18*mobsacB*_genomicRSP1566. The RSP_1566 coding sequence was deleted from pK18*mobsacB*_genomicRSP1566 by PCR with phosphorylated primers RSP_1566_deletionF and RSP_1566_deletionR. See Table S7 for primer sequences. The resulting PCR product was ligated to generate pK18*mobsacB*_ΔRSP1566 and transformed into *E. coli* DH5α. pK18*mobsacB*_ΔRSP1566 was mobilized into *R. sphaeroides* via conjugation with *E. coli* S17-1. Transconjugants were selected on LB kanamycin plates under aerobic conditions. Colonies were streaked for purity, resuspended in LB, and plated on LB sucrose (10% [wt/vol]) plates. Isolated colonies were patched onto LB kanamycin and LB sucrose plates to screen for cells that had lost the plasmid. Kanamycin-sensitive, sucrose-resistant colonies containing the desired gene deletion were identified by PCR. Gene deletion was confirmed by Sanger sequencing of the genomic region.

### Model comparison.

Parsimonious FBA (pFBA) was used to predict essential metabolic genes in *R. sphaeroides* using the constraint-based model iRsp1140 (18, 39). To simulate growth, nutrient uptake rates were set to mimic the composition of SMM, with the free exchange of phosphate, sulfate, magnesium, iron, nicotinate, thiamine, biotin, and CO_2_ permitted (i.e., upper and lower bounds of associated exchange reactions were set to 1,000 and −1,000 mmol g dry weight [gDW]^−1^ h^−1^, respectively). The uptake rates for the limiting substrates succinate and ammonium were set to 2.5 and 1 mmol gDW^−1^ h^−1^, respectively, based on previous measurements (17). For simulation of aerobic growth, the free exchange of oxygen was permitted, while the photon uptake rate was set to 0 mmol gDW^−1^ h^−1^. Conversely, for simulation of anaerobic photosynthetic growth photon uptake was unconstrained, while oxygen uptake was set to 0 mmol gDW^−1^ h^−1^. To simulate single-gene deletions under each condition, the gene-protein-reaction (GPR) Boolean logic rules within iRsp1140 were used to determine the upper and lower bounds for the associated reaction(s). For instance, if reaction A had a GPR rule consisting only of gene B, then the upper and lower bounds of reaction A are set to 0 mmol gDW^−1^ h^−1^ to simulate the impact of deleting gene B. On the other hand, if reaction A is catalyzed by multiple isozymes, the reaction bounds would be unchanged for simulating the deletion of a gene encoding one of those isozymes. Once reaction bounds had been set, FBA was performed as previously described (39). Gene deletions that resulted in a predicted biomass growth rate of 0 h^−1^ were considered essential for growth under the conditions tested. pFBA, gene essentiality determination, and reconstruction modifications were made using COBRApy (70). The Python code for modifying the original iRsp1140 SBML file is included in Data Set S1.

10.1128/mSystems.00015-17.10 DATA SET S1  Python code modifying iRsp1140. This COBRA-Py-based model updates the iRsp1140 model based on Tn-seq results. Download DATA SET S1, PDF file, 0.1 MB. Copyright © 2017 Burger et al. 2017 Burger et al. This content is distributed under the terms of the Creative Commons Attribution 4.0 International license.

### Accession number(s).

Illumina sequencing data have been deposited in the NCBI Sequence Read Archive (SRA) as BioProject PRJNA376384.
