# Supplementary material for: Combining Genome-Scale Experimental and Computational Methods To Identify Essential Genes in Rhodobacter sphaeroides
Source: mSystems. 2017 Jun 6;2(3):e00015-17. doi: 10.1128/mSystems.00015-17 (PMC5513736; doi:10.1128/mSystems.00015-17)
Supplement: TEXT S1 [file sys003172109s10.pdf]

User Guide for Tn-seq analysis software (TSAS)

by

Saheed Imam

email: saheedrimam@gmail.com

Transposon mutagenesis followed by high-throughput sequencing (Tn-seq) is a robust approach for genome-wide identification of putative essential genes in any organism of interest for which the appropriate genetic tools are available (or can be developed). However, easily accessible computational tools for the streamlined analysis of the data from Tn-seq experiments are currently limited.

This document provides detailed instructions for how to use the Tn-seq analysis software (TSAS), which provides tools for various kinds of analysis of Tn-seq data sets.

System requirements

The only system requirement for this tool is to have **Java runtime** installed and configured on your machine. Due to occasional backward compatibility issues between different versions of Java, it may be beneficial to also have Java software development kit (SDK) installed and configured to enable recompilation of the code if necessary.

Since the TSAS code is all written in Java it should be platform independent and run on any machine with Java installed. It has been tested in Linux (CentOS 6), Windows, and Mac environments with Java 1.8.

Required Data

The following input data files are required to run TSAS:

a. **Aligned reads file** obtained from mapping short reads to a reference genome. TSAS currently handles Bowtie (version 1 and 2), SOAP and Eland result formats. This could be extended in future updates.
b. **Genome sequence file** in Fasta format. The name(s) of the chromosome(s) in this file must match that used for mapping aligned reads to the reference genome.
c. **GFF v3 file** containing the coordinates of genomic elements in the reference genome. Again, the chromosome names in this file must match those in the genome sequence and aligned reads files.

Setting up a run

Once all the required data is available a TSAS run can be set up. How the run is set up will depend on the amount of data/number of conditions available and end objective. If only one condition is available (i.e., no control or reference condition), then the only option would be a one-sample analysis (see below). If both a treatment and control condition are available, then the user has the option of running a two-sample analysis. (NB: a one-sample analysis could also be run on each condition and the results compared in a post-processing step left to the user).

To set up a run, the "Parameters.txt" file first needs to be filled with the appropriate information. Below, I provide the details of the entries expected for each parameter, depending on the type of analysis being run.

The first parameter to be set in the "Parameters.txt" is the:

a. **Analysis_type:** this sets the type of analysis to be run. 1 = one-sample analysis; and 2 = two-sample analysis. The default is 1. If 2 (two-sample) is selected and no control samples are provided, the program will exit with an associated error message.

One-sample analysis

When Tn-seq data has only been collected from one condition, a one-sample analysis is run wherein the number of unique insertions in each gene is tested against a binomial distribution based on the insertion density of the entire data set (i.e., the probability of an insertion) and the length of the gene under consideration (i.e., number of trials). For this analysis, the following parameters need to be set in the "Parameters.txt" file:

a. **Genome_sequence: (Required)** Provide the absolute or relative path to the genome sequence fasta file for the target organism to be used for this analysis. If this file is in the same folder as the TSAS code, just the file name is sufficient.
b. **GFF: (Required)** Provide the absolute or relative path to the GFF v3 file for the target organism to be used for this analysis.
c. **Mapped_format: (Required)** Enter the file format of the aligned reads file to be provided. Currently only *Bowtie*, *SOAP* and *Eland* are accepted options.
d. **Treatment: (Required)** Provide the absolute or relative path to the aligned reads file(s) in the appropriate format. If there are replicates for this condition, the absolute or relative path to each file can be provided separated by commas. These replicates will be averaged prior to calculating p-values for essentiality.
e. **Min. hits:** This is the threshold for the minimum number of reads that needs to be mapped at a given insertion site before that site is accepted as a true insertion site. Default is 5. After inspecting your data by visualizing the output WIG file, increasing the min. hits to something substantially above 1 may be beneficial to get rid of background that could be the result of sample preparation or other artifacts.
f. **Clipping:** This is the percentage from the start and the end of the gene that will be ignored when assessing essentiality. Default is 5%.
g. **Result:** This determines whether the results are printed out in "Long" or "Short" format. The long format includes results from intermediate analysis like unadjusted p-values etc. Default is "Short".

Entries in the "Control", "Capping" and "Weight" parameters will be ignored in a one-sample analysis.

Once the "Parameters.txt" file has been filled, the one-sample run is started from the terminal/command line as follows:

java TSAS

Depending on the genome size and the amount of data available this command may result in an "java heap space" error, where Java essentially runs out of memory. Thus, it may be better to start the run with the following command:

java -Xmx8g TSAS

which provides Java with access to more memory (up to 8 gigs). This can be increased depending on the resources available on the machine being used for the analysis, as well as the size of the data set being analyzed.

Two-sample analysis

When Tn-seq data has been collected from both a control and treatment condition, the user has the option of running a two-sample analysis wherein the total number of reads per gene, as well as insertions per gene, is compared between samples. This enables the calculation of a fold change between the selected treatment and control conditions based on the differential abundance of reads counted in each gene (thus genes with significantly fewer reads in the treatment relative to the control may be classified as "Conditionally essential"). The two-sample analysis offers the advantage of a reference for what the expected number of insertions and reads in the gene might be under a valid control condition (i.e., a condition free of the selective pressure(s) present in the treatment condition), precluding the need to rely on any assumptions as to the true distribution of the data.

For a two-sample run, parameters (a) to (f) described in the one-sample analysis section will need to be set. In addition, the follow required or optional parameters should be specified:

h. **Control: (Required)** Provide the absolute or relative path to the aligned reads file(s) in the appropriate format for a suitable control sample. If there are replicates for this condition, the absolute or relative path to each file should be provided separated by commas. These replicates will be averaged prior to calculating p-values for essentiality.
i. **Capping:** Specify whether you would like to use the capping function to potentially mitigate any PCR artifacts in the data set. See accompanying manuscript for details of capping function. Three capping options are available to the user. Enter "1" (default) to use capping with the mean of the reads per unique insertion, plus two standard deviations. Enter "2" to use capping with the mean of the reads per unique insertion. Enter "3" to use capping with median of the reads per unique insertion site. Enter "0" for no capping.
j. **Weighting:** Specify whether you would like to use the weighting function. The weighting function is applied on a gene-level and gives greater weight to reads of genes with larger numbers of insertions. This is based on the assumption that genes that are non-essential under a given condition should have both a larger number of insertions per base pair and a large number of reads (not just the latter, which could be caused by

insertion hotspots, PCR artifacts and other processes not accounted for). See accompanying manuscript for details of weighting function. To require weighting enter “1” for this option, else enter “0”. Default is “1”.

Once the “Parameters.txt” file has been filled, the two-sample run is started from the terminal/command line as follows:

java TSAS

Additional memory can be assigned to java with the command:

java -Xmx8g TSAS

NB: The current version of the TSAS code has been tested extensively with Java 1.8. While we do not expect backward compatibility issues when earlier versions of Java runtime are used to run the code, occasions may arise where it may be necessary to recompile the code. To do this, run the following commands from the terminal/command line:

javac TSAS.java

The one- or two-sample analysis can then be run as previously described. Note that the Java software development kit (SDK) may need to be downloaded, installed and configured to recompile the code, if not previously done on your machine.

Description of results

During the course of a TSAS analysis, two or more result files are produced. These files include:

a. **WIG files:** For each aligned/mapped reads file specified, a WIG format file is produced containing the number of mapped reads per base pair for the entire genome. This file can be loaded into a genome browser, such as MochiView, to visualize the distribution of insertion sites (and number reads at each insertion site) across the genome. The WIG files are named after the aligned reads file from which the data was derived. *The WIG files will be located in the same directory as the aligned reads files*.

b. **Essential_genes.txt: (one-sample analysis only)** This file contains a list of all annotated genes in the genome along with all the statistics calculated for each gene from the data provided for a one-sample analysis. *This file will be located in the directory from which TSAS is called.* This file has the following columns:

   1. Gene ID: the unique ID for each gene obtained from the provided GFF file.
   2. Annotation: the annotation for each gene obtained from the provided GFF file.
   3. Gene length (bp): the length of each gene in base pairs.
   4. No. of Unique hits: the number of unique insertion sites for each gene.
   5. Normalize Unique hits (hits/bp): the number of unique insertion sites for each gene normalized to the length of the gene.
   6. Total number of reads: total number of reads for each gene.

7. Pvalue (Essential): The unadjusted p-values calculated from a binomial distribution assessing the likelihood of essentiality of each gene. *(Omitted from short results output)*.
8. Adj. Pvalue (Essential): p-values from column 7 corrected for multiple testing using the Benjamini-Hochberg (BH) method.
9. FWER (Essential): the family wide error rate calculated from p-values in column 7 (Bonferroni correction).
10. Pvalue (Improved fitness): The unadjusted p-values calculated from a binomial distribution assessing the likelihood of genes whose disruption by transposon insertions may result in an overall improvement of fitness. *(Omitted from short results output).*
11. Adj. Pvalue (Improved fitness): p-values from column 10 corrected for multiple testing using the BH method.
12. FWER (Improved fitness): the family wide error rate calculated from p-values in column 10 (Bonferroni correction).

c. **Conditional_essentiality.txt: (two-sample analysis only)** this file also contains a list of all annotated genes in the genome along with all the statistics calculated for each gene from a pairwise comparison of the two sample groups (conditions) provided. This file has the following columns:
1. Gene ID: the unique ID for each gene obtained from the provided GFF file.
2. Annotation: the annotation for each gene obtained from the provided GFF file.
3. AveUnique hits (treatment): the number of unique insertions per gene for the treatment condition, averaged across the replicate samples provides, if any.
4. Ave. Unique hits (control): the number of unique insertions per gene for the control condition, averaged across the replicate samples provides, if any.
5. Ave. Raw Reads(treatment): the total number of uncorrected reads per gene for the treatment condition, averaged across the replicate samples provides, if any. *(Omitted from short results output).*
6. Ave. Raw Read (control): the total number of uncorrected reads per gene for the control condition, averaged across the replicate samples provides, if any. *(Omitted from short results output).*
7. Ave. Capped_reads(treatment): the number of capped reads per gene for treatment condition, averaged across the replicate samples provides, if any. *(Omitted from short results output).*
8. Ave. Capped_reads(control): the number of capped reads per gene for control condition, averaged across the replicate samples provides, if any. *(Omitted from short results output).*
9. Ave. Weighted_reads(treatment): the number of weighted reads per gene for the treatment condition, averaged across the replicate samples provides, if any. If capping option is selected, then capped reads will be further corrected with the weighting function, if selected. If neither capping nor weighting is selected, the value in this column is equivalent to the raw reads.
10. Ave. Weighted_reads(control): the number of weighted reads per gene for the treatment condition, averaged across the replicate samples provides, if any. If capping option is selected, then capped reads will be further corrected with the weighting

function, if selected. If neither capping nor weighting is selected, the value in this column is equivalent to the raw reads.

11. Ratio_Insertions (Treatment/control): the ratio (fold change) of the total number of insertions per gene between treatment and control.
12. Log-fold Change (Insertions): $\log_2$ of the ratio value calculated in column 11.
13. Ratio_reads (Treatment/control): the ratio (fold change) of the total number of reads (or corrected reads, if appropriate options selected) per gene between treatment and control.
14. Log-fold Change: $\log_2$ of the ratio value calculated in column 13.
15. pvalue (proportions_insertions): p-values assessing the conditional essentiality of each gene based on the proportion of insertions in the treatment relative to the control condition. *(Omitted from short results output).*
16. Adj. pvalue (proportions_insertions): p-values from column 15 corrected for multiple testing using the BH method.
17. pvalue (proportions_reads): p-values assessing the conditional essentiality of each gene based on the proportion of reads in the treatment relative to the control condition. *(Omitted from short results output).*
18. Adj. pvalue (proportions_reads): p-values from column 17 corrected for multiple testing using the BH method.
19. pvalue (Fisher_insertions): p-values assessing the conditional essentiality of each gene based on fisher's exact test using insertions. *(Omitted from short results output).*
20. Adj. pvalue (Fisher_insertions): p-values from column 19 corrected for multiple testing using the BH method.
21. pvalue (Fisher_reads): p-values assessing the conditional essentiality of each gene based on fisher's exact test using reads. *(Omitted from short results output).*
22. Adj. pvalue (Fisher_reads): p-values from column 21 corrected for multiple testing using the BH method.
23. Pvalue (t-test): (optional) p-values assessing the conditional essentiality of each gene based on student's t-test. This column only appears when at least two replicates are specified for both the treatment and control conditions. *(Omitted from short results output).*
24. Adj. Pvalue (t-test): (optional) p-values from column 23 corrected for multiple testing using the BH method.
