# Supplementary figures and images for: Combining Genome-Scale Experimental and Computational Methods To Identify Essential Genes in Rhodobacter sphaeroides
Source: mSystems. 2017 Jun 6;2(3):e00015-17. doi: 10.1128/mSystems.00015-17 (PMC5513736; doi:10.1128/mSystems.00015-17)

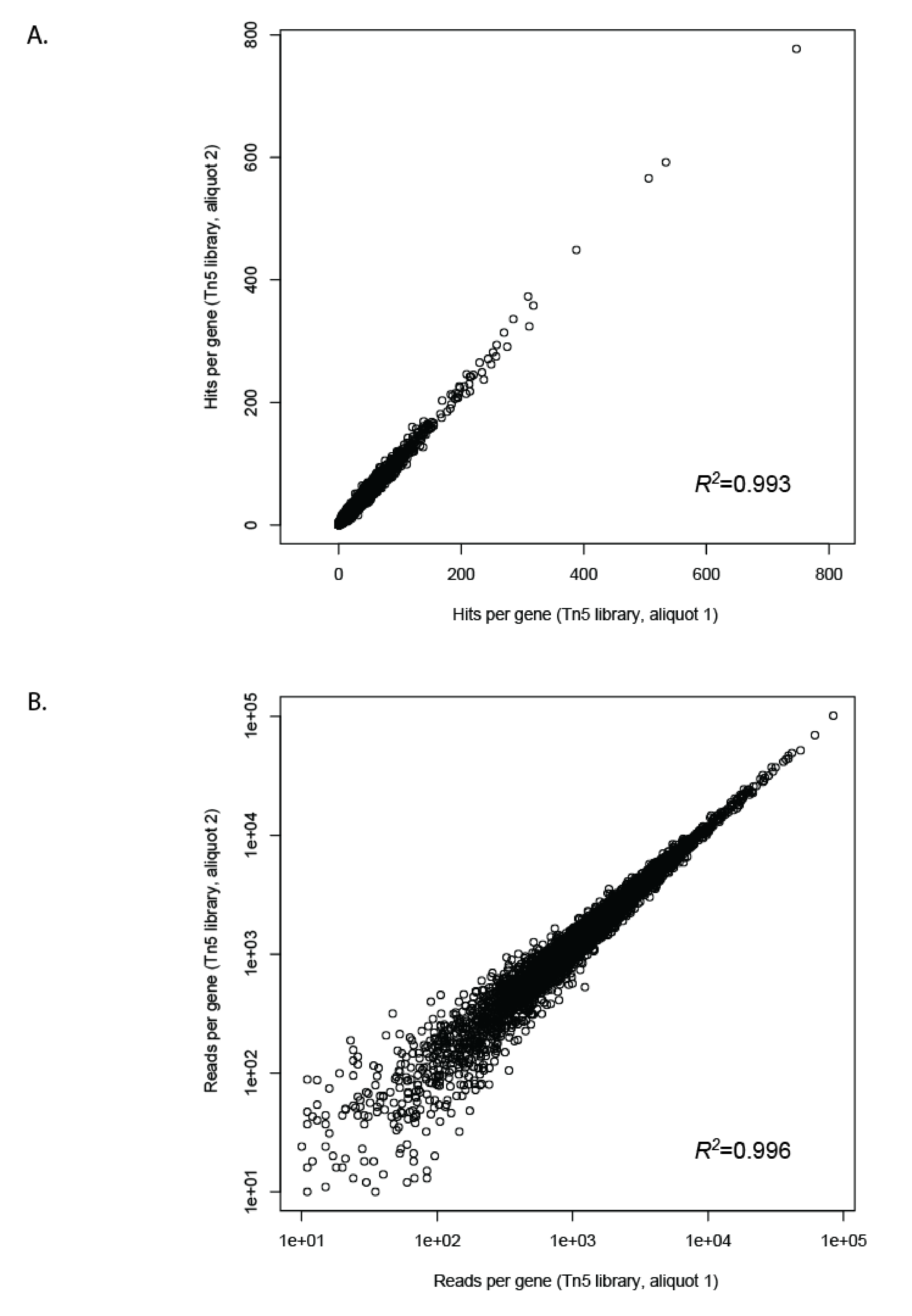

Supplement: FIG S1 [file sys003172109sf8.tif]
