# Supplementary material for: Combining Genome-Scale Experimental and Computational Methods To Identify Essential Genes in Rhodobacter sphaeroides
Source: mSystems. 2017 Jun 6;2(3):e00015-17. doi: 10.1128/mSystems.00015-17 (PMC5513736; doi:10.1128/mSystems.00015-17)
Supplement: DATA SET S1 [file sys003172109sd9.pdf]

Supplemental Data File S1 - iRsp1140_opt.py

```
#########################################
#iRsp1140vopt
#This update modifies the metabolic reconstruction of
Rhodobacter sphaeroides 2.4.1 (iRsp1140)
##and adds model constraints based on gene essentiality
determined with Tn-seq.
##Contact Matthew Scarborough (mscarborough@wisc.edu) with
questions.
#########################################

#########################################
#Import required depenencies
#########################################

from __future__ import print_function
import cobra
from cobra import Model, Reaction, Metabolite
import numpy as np
import xlsxwriter

#Import SBML downloaded from KBASE
model = cobra.io.read_sbml_model("iRsp1140.xml")

#Check number of reactions, metabolites, and genes in model
before modifications.
print("Reactions: " + str(len(model.reactions)))
print("Metabolites: " + str(len(model.metabolites)))
print("Genes: " + str(len(model.genes)))


#########################################
#Modify metabolic reconstruction
#########################################

#Modify Aerobic Biomass Reaction
#Removed Spheroidenone (0.000067693368119l807 cpd14633_c0)
#Added Vitamin B12 Coenzyme (0.0000676933681191807 cpd00166_c0)

reaction = Reaction('Biomass_Aero')
reaction.name = 'Biomass_Aero'
reaction.subsystem = ''
reaction.lower_bound = 0.  # This is the default
reaction.upper_bound = 1000.  # This is the default
reaction.objective_coefficient = 0. # this is the default
```

```
model.add_reaction(reaction)

reaction.reaction = "48.8795598256 cpd00002_c0 +
0.817579349929507 cpd11770_c0 + 0.430368015578729 cpd12036_c0 +
0.000059 cpd00042_c0 + 0.0000676933681191807 cpd00166_c0 +
0.142057409042004 cpd12313_c0 + 0.333864881166228 cpd12226_c0 +
0.071328403696196 cpd12255_c0 + 0.16003935955365 cpd12227_c0 +
0.400398098059319 cpd12060_c0 + 0.552645279057922 cpd12100_c0 +
0.136063425538122 cpd12228_c0 + 0.29010880158789 cpd12256_c0 +
0.550247685656369 cpd12003_c0 + 0.16663274140792 cpd01326_c0 +
0.158241164502485 cpd12105_c0 + 0.203795439131989 cpd12335_c0 +
0.323675109209629 cpd12164_c0 + 0.27332564777702 cpd12132_c0 +
0.312885938902641 cpd12229_c0 + 0.0629368267907612 cpd12336_c0 +
0.12047906842802 9  cpd12194_c0 + 0.451946356192704 cpd12133_c0 +
0.093471857028361 cpd00038_c0 + 0.0907387617936135 cpd00052_c0 +
0.0437295237559583 cpd00062_c0 + 0.0185403408113183 cpd00115_c0
+ 0.0185642947400151 cpd00241_c0+ 0.0411528495010915 cpd00356_c0
+ 0.0414882045028466 cpd00357_c0 + 0.027400 cpd13401_c0 +
0.00834 cpd11649_c0 + 0.077027 cpd11456_c0 + 0.086655
cpd11652_c0 + 0.038513 cpd11624_c0 + 0.002407 cpd12801_c0 +
0.024071 cpd14231_c0 + 0.002140 cpd00003_c0 + 0.00005
cpd00004_c0 + 0.000129 cpd00006_c0 + 0.000398 cpd00005_c0 +
0.000003 cpd00078_c0 + 0.00005 cpd00022_c0 + 0.000059
cpd00010_c0 + 0.00001 cpd00015_c0 + 0.049752 cpd00345_c0 +
0.000995 cpd00018_c0 + 0.002990 cpd00089_c0 + 0.034830
cpd00118_c0 + 0.006970 cpd00264_c0 + 0.000059 cpd00017_c0 +
0.000059 cpd00056_c0 + 0.000059 cpd00016_c0 + 0.000059
cpd11313_c0 + 0.000059 cpd00220_c0 + 0.000059 cpd02229_c0 +
0.000059 cpd11665_c0 + 0.000059 cpd03517_c0 + 48.8340638966
cpd00001_c0 => 48.8340638966 cpd00008_c0 + 48.8340638966
cpd00009_c0 + 48.8340638966 cpd00067_c0 + 0.284470 cpd00012_c0 +
0.817579349929507 cpd11906_c0 + 0.430368015578729 cpd11907_c0 +
0.142057409042004 cpd11908_c0 + 0.333864881166228 cpd11909_c0 +
0.071328403696196 cpd11910_c0 + 0.16003935955365 cpd11912_c0 +
0.400398098059319 cpd11911_c0 + 0.552645279057922 cpd11913_c0 +
0.136063425538122 cpd11914_c0 + 0.29010880158789 cpd11915_c0 +
0.550247685656369 cpd11916_c0 + 0.16663274140792 cpd11917_c0 +
0.158241164502485 cpd11918_c0 + 0.203795439131989 cpd11919_c0 +
0.323675109209629 cpd11920_c0 + 0.27332564777702 cpd11921_c0 +
0.312885938902641 cpd11922_c0 + 0.0629368267907612 cpd11923_c0 +
0.120479068428029 cpd11751_c0 + cpd11416_c0 + 0.451946356192704
cpd11924_c0"

#Modify Photo Biomass Reaction
#Added Vitamin B12 Coenzyme (0.0000676933681191807 cpd00166_c0)
#Added Pyridoxal 5-Phosphate (0.000059 cpd00016_c0)
```

```
reaction = Reaction('Biomass_Photo')
reaction.name = 'Biomass_Photo'
reaction.subsystem = ''
reaction.lower_bound = 0.  # This is the default
reaction.upper_bound = 1000.  # This is the default
reaction.objective_coefficient = 0. # this is the default

model.add_reaction(reaction)

reaction.reaction = "0.000059 cpd00016_c0 +
0.0000676933681191807 cpd00166_c0 + 0.0105866653788 cpd00241_c0
+ 0.000059 cpd00220_c0 + 0.00142908221585 cpd14630_c0 + 0.03483
cpd00118_c0 + 0.00001 cpd00015_c0 + 0.407290710076 cpd12060_c0 +
0.00697 cpd00264_c0 + 0.000059 cpd00056_c0 + 0.0640202463443
cpd12336_c0 + 0.295102849816 cpd12256_c0 + 0.100182 cpd11652_c0
+ 0.0236594895949 cpd00357_c0 + 0.00214 cpd00003_c0 +
0.00317621466582 cpd08107_c0 + 0.144502841749 cpd12313_c0 +
0.0234682466074 cpd00356_c0 + 0.00005 cpd00004_c0 + 0.044525
cpd11624_c0 + 0.0631160449388 cpd00038_c0 + 0.000129 cpd00006_c0
+ 0.00299 cpd00089_c0 + 0.562158734566 cpd12100_c0 +
0.339612163941 cpd12226_c0 + 0.437776541669 cpd12036_c0 +
0.122553043002 cpd12194_c0 + 0.0105730051654 cpd00115_c0 +
0.027828 cpd14231_c0 + 0.00834 cpd11649_c0 + 0.000003
cpd00078_c0 + 0.00005 cpd00022_c0 + 0.0274 cpd13401_c0 +
0.831653485844 cpd11770_c0 + 0.002783 cpd12801_c0 + 0.000059
cpd00017_c0 + 53.64655271 cpd00002_c0 + 0.207303654829
cpd12335_c0 + 0.0725562791902 cpd12255_c0 + 0.000059 cpd11313_c0
+ 0.000398 cpd00005_c0 + 0.0612705465487 cpd00052_c0 + 0.08905
cpd11456_c0 + 0.0295279742404 cpd00062_c0 + 0.278030784124
cpd12132_c0 + 0.169501223654 cpd01326_c0 + 0.000059 cpd03517_c0
+ 0.160965190808 cpd12105_c0 + 0.13840567543 cpd12228_c0 +
0.000995 cpd00018_c0 + 0.000059 cpd03444_c0 + 0.000059
cpd00042_c0 + 0.000059 cpd02229_c0 + 0.559719868039 cpd12003_c0
+ 0.162794340704 cpd12227_c0 + 0.459726340415 cpd12133_c0 +
0.000059 cpd00010_c0 + 0.329246981199 cpd12164_c0 + 0.049752
cpd00345_c0 + 0.318272081826 cpd12229_c0 --> 0.407290710076
cpd11911_c0 + 0.437776541669 cpd11907_c0 + 0.339612163941
cpd11909_c0 + 0.562158734566 cpd11913_c0 + 0.144502841749
cpd11908_c0 + 0.169501223654 cpd11917_c0 + cpd11416_c0 + 0.28447
cpd00012_c0 + 0.207303654829 cpd11919_c0 + 0.295102849816
cpd11915_c0 + 53.615732887 cpd00008_c0 + 0.0725562791902
cpd11910_c0 + 0.329246981199 cpd11920_c0 + 0.122553043002
cpd11751_c0 + 0.160965190808 cpd11918_c0 + 0.318272081826
cpd11922_c0 + 0.278030784124 cpd11921_c0 + 0.831653485844
cpd11906_c0 + 0.559719868039 cpd11916_c0 + 0.13840567543
cpd11914_c0 + 53.615732887 cpd00009_c0 + 0.0640202463443
cpd11923_c0 + 53.615732887 cpd00001_c0 + 0.162794340704
```

```
cpd11912_c0 + 53.615732887 cpd00067_c0 + 0.459726340415
cpd11924_c0"

#Change GPR for hcys-L_c0 + 5mthf_c0 <=> met-L_c0 + h_c0 +
thf_c0
reaction = model.reactions.get_by_id("RXN0155_c0")
reaction.gene_reaction_rule = ("RSP_3346 and RSP_3347")

#Change GPR for f6p_c0 + g3p_c0 <=> xu5p-D_c0 + e4p_c0
reaction = model.reactions.get_by_id("RXN0553_c0")
reaction.gene_reaction_rule = ("RSP_2956")

#Change GPR for g3p_c0 + s7p_c0 <=> r5p_c0 + xu5p-D_c0
reaction = model.reactions.get_by_id("RXN0554_c0")
reaction.gene_reaction_rule = ("RSP_2956")

#Change GPR for uaagmda_c0 --> [GlcNAc-(1->4)-Mur2Ac(oyl-L-Ala-
g-D-Glu-A2pm-D-Ala-D-Ala)]n- diphosphoundecaprenol_c0 +
udcpdp_c0
reaction = model.reactions.get_by_id("RXN0572_c0")
reaction.gene_reaction_rule = ("RSP_2326 and RSP_2098 and
RSP_0550")

#Change GPR for 23dhmb_c0 --> 3mob_c0 + h2o_c0
reaction = model.reactions.get_by_id("RXN1040_c0")
reaction.gene_reaction_rule = ("RSP_3074")

#Change GPR for f6p_c0 + gln-L_c0 <=> glu-L_c0 + gam6p_c0
reaction = model.reactions.get_by_id("RXN0046_c0")
reaction.gene_reaction_rule = ("RSP_1447 and RSP_2502")

#Change GPR for gly_c0 + succoa_c0 + h_c0 --> co2_c0 + 5aop_c0 +
coa_c0
reaction = model.reactions.get_by_id("RXN0621_c0")
reaction.gene_reaction_rule = ("RSP_2984")

#Change GPR for co2_c0 + alac-S_c0 <=> 2.0 pyr_c0 + h_c0
reaction = model.reactions.get_by_id("RXN1033_c0")
reaction.gene_reaction_rule = ("RSP_2637 and (RSP_2636 or
RSP_3284 or RSP_3155)")

#Set flux through redundant Valine synthesis reactions to zero.
#co2_c0 + CPD0544_c0 <=> 2.0 pyr_c0 + h_c0
rxn=model.reactions.get_by_id('RXN1034_c0')
rxn.delete()

#CPD1037_c0 + nadp_c0 <=> h_c0 + CPD0544_c0 + nadph_c0
```

```
rxn=model.reactions.get_by_id('RXN1037_c0')
rxn.delete()

#CPD1037_c0 <=> h2o_c0 + 3mob_c0
rxn=model.reactions.get_by_id('RXN1046_c0')
rxn.delete()

#4mop_c0 + lpam_c0 + h_c0 <=> co2_c0 + S-(3-Methylbutanoyl)-
dihydrolipoamide-E_c0
rxn=model.reactions.get_by_id('RXN1053_c0')
rxn.lower_bound = 0
rxn.upper_bound = 1000

#Change GPR for coa_c0 + 3c3hmp_c0 + h_c0 <=> 3mob_c0 + h2o_c0 +
accoa_c0
reaction = model.reactions.get_by_id("RXN0828_c0")
reaction.gene_reaction_rule = ("RSP_2723")

#Change GPR for trnalys_c0 + atp_c0 + lys-L_c0 --> ppi_c0 +
lystrna_c0 + amp_c0
reaction = model.reactions.get_by_id("RXN0067_c0")
reaction.gene_reaction_rule = "RSP_0584 and (RSP_0478 or
RSP_4329)"

#Change GPR for trnahis_c0 + his-L_c0 + atp_c0 --> ppi_c0 +
histrna_c0 + amp_c0
reaction = model.reactions.get_by_id("RXN0070_c0")
reaction.gene_reaction_rule = "RSP_3550 and RSP_3551"

#Change GPR for atp_c0 + trnaglu_c0 + glu-L_c0 --> ppi_c0 +
glutrna_c0 + amp_c0
reaction = model.reactions.get_by_id("RXN0053_c0")
reaction.gene_reaction_rule = "RSP_0797 and RSP_1995 "

#Change GPR for pi_c0 + adp_c0 + 4.0 h_p0 <=> atp_c0 + 3.0 h_c0
+ h2o_c0
reaction = model.reactions.get_by_id("RXN0520_c0")
reaction.gene_reaction_rule = "RSP_2296 and RSP_2297 and
RSP_2299 and RSP_2298 and RSP_1038 and (RSP_1035 or RSP_1036 or
RSP_7012)"

#Change GPR for atp_c0 + ASPARTYL-TRNAASN_c0 + h2o_c0 + gln-L_c0
--> pi_c0 + asntrna_c0 + adp_c0 + glu-L_c0
reaction = model.reactions.get_by_id("RXN0054_c0")
reaction.gene_reaction_rule = "RSP_2270 and RSP_1986 and
RSP_3369"
```

```
#Change GPR for gln-L_c0 + glutrna(gln)_c0 + atp_c0 + h2o_c0 -->
pi_c0 + glntrna_c0 + adp_c0 + glu-L_c0
reaction = model.reactions.get_by_id("RXN0055_c0")
reaction.gene_reaction_rule = "RSP_2270 and RSP_1986 and
RSP_3369"

#Change GPR for argsuc_c0 <=> fum_c0 + arg-L_c0
reaction = model.reactions.get_by_id("RXN0092_c0")
reaction.gene_reaction_rule = "(RSP_0726 and RSP_3955)"

#Change reversibility of 2.0 glu-L_c0 + nadp_c0 <=> gln-L_c0 +
akg_c0 + h_c0 + nadph_c0 to operate in reverse only.
rxn = model.reactions.get_by_id("RXN0008_c0")
rxn.lower_bound = -1000
rxn.upper_bound = 0

#Change GPR for atp_c0 + nh4_c0 + glu-L_c0 --> pi_c0 + gln-L_c0
+ adp_c0 + h_c0
reaction = model.reactions.get_by_id("RXN0006_c0")
reaction.gene_reaction_rule = "RSP_0147"

#Change GPR for nad_c0 + pyr_c0 + coa_c0 --> nadh_c0 + accoa_c0
+ co2_c0
reaction = model.reactions.get_by_id("RXN0421_c0")
reaction.gene_reaction_rule = "RSP_4047 and RSP_4049 and
RSP_4050 and RSP_2968 and RSP_0962"

#Make thr-L_c0 + nad_c0 <=> nadh_c0 + 2aobut_c0 + h_c0
irreversible
rxn = model.reactions.get_by_id("RXN0399_c0")
rxn.lower_bound = 0
rxn.upper_bound = 1000

#Set flux through qh2_c0 + 2.0 h_c0 + 0.5 o2_c0 --> q_c0 + 2.0
h_p0 + h2o_c0 to 0.
rxn = model.reactions.get_by_id("RXN0519_c0")
rxn.lower_bound = 0
rxn.upper_bound = 0

#Make gln-L_c0 + atp_c0 + h2o_c0 + xmp_c0 <=> gmp_c0 + ppi_c0 +
2.0 h_c0 + glu-L_c0 + amp_c0 irreversible.
rxn = model.reactions.get_by_id("RXN0746_c0")
rxn.lower_bound = 0
rxn.upper_bound = 1000

#Change reversibility of ppi_c0 + xmp_c0 <=> prpp_c0 + xan_c0
operate in reverse only.
```

```
rxn = model.reactions.get_by_id("RXN0745_c0")
rxn.lower_bound = -1000
rxn.upper_bound = 0

#Change GPR for dhor-S_c0 + h2o_c0 <=> cbasp_c0 + h_c0
reaction = model.reactions.get_by_id("RXN0788_c0")
reaction.gene_reaction_rule = "RSP_1003 and RSP_1930"

#Change GPR for 2.0 glu-L_c0 + nadp_c0 <=> gln-L_c0 + akg_c0 +
h_c0 + nadph_c0
reaction = model.reactions.get_by_id("RXN0008_c0")
reaction.gene_reaction_rule = "RSP_1149"

#butACP_c0 + malACP_c0 <=> ACP_c0 + co2_c0 + 3oxhACP_c0
reaction = model.reactions.get_by_id("RXN0273_c0")
reaction.gene_reaction_rule = "RSP_3177"

#hexACP_c0 + malACP_c0 <=> ACP_c0 + co2_c0 + 3oxocACP_c0
reaction = model.reactions.get_by_id("RXN0274_c0")
reaction.gene_reaction_rule = "RSP_3177"

#octACP_c0 + malACP_c0 <=> ACP_c0 + co2_c0 + 3oxdeACP_c0
reaction = model.reactions.get_by_id("RXN0275_c0")
reaction.gene_reaction_rule = "RSP_3177"

#decACP_c0 + malACP_c0 <=> ACP_c0 + co2_c0 + 3oxddACP_c0
reaction = model.reactions.get_by_id("RXN0276_c0")
reaction.gene_reaction_rule = "RSP_3177"

#ddcaACP_c0 + malACP_c0 <=> ACP_c0 + co2_c0 + 3oxtdACP_c0
reaction = model.reactions.get_by_id("RXN0277_c0")
reaction.gene_reaction_rule = "RSP_3177"

#myrsACP_c0 + malACP_c0 <=> 3oxhdACP_c0 + co2_c0 + ACP_c0
reaction = model.reactions.get_by_id("RXN0278_c0")
reaction.gene_reaction_rule = "RSP_2464"

#hedacp_c0 + malACP_c0 <=> ACP_c0 + co2_c0 + 3oxocdacp_c0
reaction = model.reactions.get_by_id("RXN0279_c0")
reaction.gene_reaction_rule = "RSP_2464"

#nadp_c0 + 3hbACP_c0 <=> actACP_c0 + h_c0 + nadph_c0
reaction = model.reactions.get_by_id("RXN0263_c0")
reaction.gene_reaction_rule = "RSP_2461"

#3hhACP_c0 + nadp_c0 <=> h_c0 + 3oxhACP_c0 + nadph_c0
reaction = model.reactions.get_by_id("RXN0264_c0")
```

```
reaction.gene_reaction_rule = "RSP_2461"

#nadp_c0 + 3hocACP_c0 <=> h_c0 + nadph_c0 + 3oxocACP_c0
reaction = model.reactions.get_by_id("RXN0265_c0")
reaction.gene_reaction_rule = "RSP_2461"

#3hdeACP_c0 + nadp_c0 <=> h_c0 + nadph_c0 + 3oxdeACP_c0
reaction = model.reactions.get_by_id("RXN0266_c0")
reaction.gene_reaction_rule = "RSP_2461"

#3htdACP_c0 + nadp_c0 <=> 3oxtdACP_c0 + h_c0 + nadph_c0
reaction = model.reactions.get_by_id("RXN0267_c0")
reaction.gene_reaction_rule = "RSP_2461"

#nadp_c0 + 3hddACP_c0 <=> h_c0 + nadph_c0 + 3oxddACP_c0
reaction = model.reactions.get_by_id("RXN0268_c0")
reaction.gene_reaction_rule = "RSP_2461"

#3hpaACP_c0 + nadp_c0 <=> 3oxhdACP_c0 + h_c0 + nadph_c0
reaction = model.reactions.get_by_id("RXN0269_c0")
reaction.gene_reaction_rule = "RSP_2461"

#nadph_c0 + 3oxocdacp_c0 + h_c0 <=> 3hc18acp_c0 + nadp_c0
reaction = model.reactions.get_by_id("RXN0270_c0")
reaction.gene_reaction_rule = "RSP_2461"

#Change GPR for 3hdeACP_c0 <=> 2tdeACP_c0 + h2o_c0
reaction = model.reactions.get_by_id("RXN0280_c0")
reaction.gene_reaction_rule = "RSP_2172 and RSP_3178"

#Change GPR for RXN0281_c0   3hbACP_c0 <=> 2beACP_c0 + h2o_c0
reaction = model.reactions.get_by_id("RXN0281_c0")
reaction.gene_reaction_rule = "RSP_2172 and RSP_3178"

#Change GPR for RXN0282_c0   3hhACP_c0 <=> h2o_c0 + 2theACP_c0
reaction = model.reactions.get_by_id("RXN0282_c0")
reaction.gene_reaction_rule = "RSP_2172 and RSP_3178"

#Change GPR for RXN0283_c0 3hocACP_c0 <=> 2toceACP_c0 + h2o_c0
reaction = model.reactions.get_by_id("RXN0283_c0")
reaction.gene_reaction_rule = "RSP_2172 and RSP_3178"

#Change GPR for RXN0285_c0 3hddACP_c0 <=> 2tddACP_c0 + h2o_c0
reaction = model.reactions.get_by_id("RXN0285_c0")
reaction.gene_reaction_rule = "RSP_2172 and RSP_3178"

#Change GPR for RXN0286_c0 3htdACP_c0 <=> h2o_c0 + tmrs2eACP_c0
```

```
reaction = model.reactions.get_by_id("RXN0286_c0")
reaction.gene_reaction_rule = "RSP_2172 and RSP_3178"

#Change GPR for RXN0287_c0 3hpaACP_c0 <=> tpalm2eACP_c0 + h2o_c0
reaction = model.reactions.get_by_id("RXN0287_c0")
reaction.gene_reaction_rule = "RSP_2172 and RSP_3178"

#Change GPR for RXN0288_c0 3hc18acp_c0 <=> tocdeenacp_c0 +
h2o_c0
reaction = model.reactions.get_by_id("RXN0288_c0")
reaction.gene_reaction_rule = "RSP_2172 and RSP_3178"

#Change GPR for 3ig3p_c0 + ser-L_c0 <=> h2o_c0 + g3p_c0 + trp-
L_c0
reaction = model.reactions.get_by_id("RXN0605_c0")
reaction.gene_reaction_rule = "RSP_0831 and RSP_3585"

#Change GPR for ser-L_c0 + indole_c0 --> h2o_c0 + trp-L_c0
reaction = model.reactions.get_by_id("RXN0604_c0")
reaction.gene_reaction_rule = "RSP_0831 and RSP_3585"

#Make arg-L_c0 + h2o_c0 <=> orn_c0 + urea_c0 irreversible
rxn = model.reactions.get_by_id("RXN0075_c0")
rxn.lower_bound = 0
rxn.upper_bound = 1000

#Change GPR for ckdo_c0 + lipid4A_c0 <=> cmp_c0 + 3.0 h_c0 +
kdolipid4_c0
reaction = model.reactions.get_by_id("RXN0459_c0")
reaction.gene_reaction_rule = "RSP_1461 and RSP_2394"

#Chaneg GPR for h_c0 + o2_c0 + 2ommb_c0 + nadph_c0 -->
OCTAPRENYL-METHYL-OH-METHOXY-BENZQ_c0 + h2o_c0 + nadp_c0
reaction = model.reactions.get_by_id("RXN1026_c0")
reaction.gene_reaction_rule = "RSP_1869 and RSP_1492"

#Change GPR for nadph_c0 + 2omph_c0 + o2_c0 --> h_c0 + nadp_c0 +
h2o_c0 + 2ombz_c0
reaction = model.reactions.get_by_id("RXN1024_c0")
reaction.gene_reaction_rule = "RSP_1869 and RSP_1492"

#Change GPR for sl26da_c0 + akg_c0 <=> glu-L_c0 + sl2a6o_c0
reaction = model.reactions.get_by_id("RXN0099_c0")
reaction.gene_reaction_rule = "RSP_2008 and RSP_1494"

#Change GPR for "sl26da_c0 + h2o_c0 <=> 26dap-LL_c0 + succ_c0"
reaction = model.reactions.get_by_id("RXN0467_c0")
```

```
reaction.gene_reaction_rule = "RSP_1128"

#Change GPR for "glyc3p_c0 + acoa_c0 <=> 1ag3p_c0 + coa_c0"
reaction = model.reactions.get_by_id("RXN0363_c0")
reaction.gene_reaction_rule = "RSP_2613 and RSP_1004"

#Change GPR for ru5p-D_c0 --> for_c0 + db4p_c0
reaction = model.reactions.get_by_id("RXN0829_c0")
reaction.gene_reaction_rule = "RSP_0846 and RSP_0757"

#Change GPR for gtp_c0 + 3.0 h2o_c0 --> ppi_c0 + 25drapp_c0 +
for_c0 + h_c0
reaction = model.reactions.get_by_id("RXN0830_c0")
reaction.gene_reaction_rule = "RSP_0846 and RSP_0757"

#Make orn_c0 <=> nh4_c0 + pro-L_c0 irreversible
rxn = model.reactions.get_by_id("RXN0074_c0")
rxn.lower_bound = 0
rxn.upper_bound = 1000

#Change GPR for h2s_c0 + suchms_c0 <=> succ_c0 + h_c0 + hcys-
L_c0
reaction = model.reactions.get_by_id("RXN0163_c0")
reaction.gene_reaction_rule = "RSP_1851"

#Remove accoa_c0 + hom-L_c0 <=> achms_c0 + coa_c0
rxn=model.reactions.get_by_id('RXN1403_c0')
rxn.delete()

#Delete h2o_c0 + o2_c0 + so3_c0 <=> so4_c0 + h2o2_c0
#Deleting reaction originally results in no growth.

rxn=model.reactions.get_by_id('RXN1311_c0')
rxn.lower_bound = 0
rxn.upper_bound = 0

#Remove nadp_c0 + h2o_c0 + glu5sa_c0 <=> 2.0 h_c0 + glu-L_c0 +
nadph_c0
rxn=model.reactions.get_by_id('RXN0009_c0')
rxn.delete()

#Make nadp_c0 + 2.0 h2o_c0 + 1pyr5c_c0 <=> h_c0 + nadph_c0 +
glu-L_c0 irreversible
rxn=model.reactions.get_by_id('RXN0010_c0')
rxn.lower_bound = 0
rxn.upper_bound = 1000
```

```
#Set flux through "orn_c0 --> nh4_c0 + pro-L_c0" to zero
rxn=model.reactions.get_by_id('RXN0074_c0')
rxn.lower_bound = 0
rxn.upper_bound = 0

#Change GPR for accoa_c0 + btn_co2_c0 + h_c0 <=> btn_cbxl_c0 +
malcoa_c0
reaction=model.reactions.get_by_id("RXN0271_c0")
reaction.gene_reaction_rule = "RSP_1772 and RSP_0929"

#Change GPR for atp_c0 + asp-L_c0 + 5aizc_c0 <=> pi_c0 + adp_c0
+ h_c0 + 25aics_c0
reaction=model.reactions.get_by_id("RXN0707_c0")
reaction.gene_reaction_rule = "RSP_2126 and RSP_2258"

#Change GPR for akg_c0 + hisp_c0 <=> glu-L_c0 + imacp_c0
reaction=model.reactions.get_by_id("RXN0438_c0")
reaction.gene_reaction_rule = "RSP_2284"

#ppi_e0 <=> ppi_p0
rxn=model.reactions.get_by_id('RXN1676_c0')
rxn.lower_bound = 0
rxn.upper_bound = 1000

#Make ala-D_c0 + akg_c0 <=> pyr_c0 + glu-D_c0 irreversible
rxn=model.reactions.get_by_id("RXN0170_c0")
rxn.lower_bound = 0
rxn.upper_bound = 1000

#Make dump_c0 + mlthf_c0 <=> dtmp_c0 + dhf_c0 irreversible
rxn=model.reactions.get_by_id("RXN0805_c0")
rxn.lower_bound = 0
rxn.upper_bound = 1000

#Change GPR for akg_c0 + hisp_c0 <=> glu-L_c0 + imacp_c0
reaction=model.reactions.get_by_id("RXN0464_c0")
reaction.gene_reaction_rule = "RSP_0882"

#Change GPR for akg_c0 + ile-L_c0 <=> glu-L_c0 + 3mop_c0
reaction=model.reactions.get_by_id("RXN1041_c0")
reaction.gene_reaction_rule = "RSP_1140"

#Change GPR for leu-L_c0 + akg_c0 <=> 4mop_c0 + glu-L_c0
reaction=model.reactions.get_by_id("RXN1042_c0")
reaction.gene_reaction_rule = "RSP_1140"

#Change GPR for akg_c0 + val-L_c0 <=> 3mob_c0 + g lu-L_c0
```

```
reaction=model.reactions.get_by_id("RXN1043_c0")
reaction.gene_reaction_rule = "RSP_1140"

#Remove fald_c0 + 3mob_c0 --> 2dhp_c0
rxn=model.reactions.get_by_id('RXN0523_c0')
rxn.delete()

#Change GPR for h2o_c0 + 3mob_c0 + mlthf_c0 --> 2dhp_c0 + thf_c0
reaction=model.reactions.get_by_id("RXN1443_c0")
reaction.gene_reaction_rule = "RSP_1756"

#nadh_c0 + 2beACP_c0 + h_c0 --> nad_c0 + butACP_c0
reaction=model.reactions.get_by_id("RXN0239_c0")
reaction.gene_reaction_rule = "RSP_3176 and RSP_2344"

#nadh_c0 + 2theACP_c0 + h_c0 --> nad_c0 + hexACP_c0
reaction=model.reactions.get_by_id("RXN0240_c0")
reaction.gene_reaction_rule = "RSP_3176 and RSP_2344"

#nadh_c0 + 2toceACP_c0 + h_c0 --> nad_c0 + octACP_c0
reaction=model.reactions.get_by_id("RXN0241_c0")
reaction.gene_reaction_rule = "RSP_3176 and RSP_2344"

#nadh_c0 + h_c0 + 2tdeACP_c0 --> nad_c0 + decACP_c0
reaction=model.reactions.get_by_id("RXN0242_c0")
reaction.gene_reaction_rule = "RSP_3176 and RSP_2344"

#nadh_c0 + h_c0 + 2tddACP_c0 --> nad_c0 + ddcaACP_c0
reaction=model.reactions.get_by_id("RXN0243_c0")
reaction.gene_reaction_rule = "RSP_3176 and RSP_2344"

#nadh_c0 + tmrs2eACP_c0 + h_c0 --> nad_c0 + myrsACP_c0
reaction=model.reactions.get_by_id("RXN0244_c0")
reaction.gene_reaction_rule = "RSP_3176 and RSP_2344"

#nadh_c0 + h_c0 + tpalm2eACP_c0 --> nad_c0 + hedacp_c0
reaction=model.reactions.get_by_id("RXN0245_c0")
reaction.gene_reaction_rule = "RSP_3176 and RSP_2344"

#nadh_c0 + h_c0 + tocdeenacp_c0 --> nad_c0 + octadec-acp_c0
reaction=model.reactions.get_by_id("RXN0246_c0")
reaction.gene_reaction_rule = "RSP_3176 and RSP_2344"

#Make cdpdag_c0 + chol_c0 <=> pc_SC_c0 + h_c0 + cmp_c0
irreversible
rxn=model.reactions.get_by_id("RXN0384_c0")
rxn.lower_bound = 0
```

```
rxn.upper_bound = 1000

#Change GPR for fdp_c0 <=> dhap_c0 + g3p_c0
reaction=model.reactions.get_by_id("RXN0555_c0")
reaction.gene_reaction_rule = "RSP_4045"

#Change GPR for s17bp_c0 <=> e4p_c0 + dhap_c0
reaction=model.reactions.get_by_id("RXN0571_c0")
reaction.gene_reaction_rule = "RSP_4045"

#Make hmgcoa_c0 <=> acac_c0 + accoa_c0 + h_c0 irreversible
rxn=model.reactions.get_by_id("RXN0868_c0")
rxn.lower_bound = 0
rxn.upper_bound = 1000

#Change GPR for ump_c0 + atp_c0 <=> udp_c0 + adp_c0
reaction=model.reactions.get_by_id("RXN0794_c0")
reaction.gene_reaction_rule = "RSP_2705"

#Change GPR for accoa_c0 + ser-L_c0 --> acser_c0 + coa_c0
reaction=model.reactions.get_by_id("RXN0153_c0")
reaction.gene_reaction_rule = "RSP_2481"

#Change GPR for g3p_c0 + nad_c0 + pi_c0 <=> 13dpg_c0 + nadh_c0 +
h_c0
reaction=model.reactions.get_by_id("RXN0417_c0")
reaction.gene_reaction_rule = "RSP_3269"

#Change GPR for nadp_c0 + 5mthf_c0 <=> 2.0 h_c0 + mlthf_c0 +
nadph_c0
reaction=model.reactions.get_by_id("RXN0477_c0")
reaction.gene_reaction_rule = "RSP_2172"

#Change GPR for nad_c0 + phthr_c0 --> nadh_c0 + co2_c0 + 1-
AMINO-PROPAN-2-ONE-3-PHOSPHATE_c0
reaction=model.reactions.get_by_id("RXN1069_c0")
reaction.gene_reaction_rule = "RSP_2904"

#Change GPR for h_c0 + uppg3_c0 + 2.0 amet_c0 <=> pre2_c0 + 2.0
ahcys_c0
reaction=model.reactions.get_by_id("RXN0640_c0")
reaction.gene_reaction_rule = "RSP_1944"

#Change GPR for 56dura_c0 + nadp_c0 <=> nadph_c0 + ura_c0 + h_c0
reaction=model.reactions.get_by_id("RXN1076_c0")
reaction.gene_reaction_rule = "RSP_0187 or RSP_0189"
```

```
#Change GPR for 3ophb_c0 + h_c0 --> 2oph_c0 + co2_c0
reaction=model.reactions.get_by_id("RXN1022_c0")
reaction.gene_reaction_rule = "RSP_0467 or RSP_0468"

#Change GPR for cdpdag_c0 + h_c0 + pg_c0 <=> cmp_c0 + cdlp_c0
reaction=model.reactions.get_by_id("RXN1352_c0")
reaction.gene_reaction_rule = "RSP_0473 or RSP_0113"

#Change GPR for gln-L_c0 + chor_c0 <=> glu-L_c0 + 4adcho_c0
reaction=model.reactions.get_by_id("RXN0334_c0")
reaction.gene_reaction_rule = "RSP_0817 or RSP_0244 or RSP_1513"

#Change GPR for atp_c0 + h2o_c0 + mn2_p0 --> mn2_c0 + adp_c0 +
h_c0 + pi_c0
reaction=model.reactions.get_by_id("RXN0937_c0")
reaction.gene_reaction_rule = "RSP_0904 and RSP_0906 and
RSP_0908"

#Change GPR for atp_c0 + fe2_p0 + h2o_c0 --> adp_c0 + h_c0 +
fe2_c0 + pi_c0
reaction=model.reactions.get_by_id("RXN0938_c0")
reaction.gene_reaction_rule = "RSP_0904 and RSP_0906 and
RSP_0908"

#Change gpr for 2.0 na1_p0 + mal-D_p0 <=> 2.0 na1_c0 + mal-D_c0
reaction=model.reactions.get_by_id("RXN1154_c0")
reaction.gene_reaction_rule = "RSP_0910"

#Change gpr for succ_p0 + 2.0 na1_p0 --> succ_c0 + 2.0 na1_c0
reaction=model.reactions.get_by_id("RXN0904_c0")
reaction.gene_reaction_rule = "RSP_0910"

#Change gpr for mal-L_p0 + 2.0 na1_p0 <=> 2.0 na1_c0 + mal-L_c0
reaction=model.reactions.get_by_id("RXN0905_c0")
reaction.gene_reaction_rule = "RSP_0910"

#Change gpr for fum_p0 + 2.0 na1_p0 <=> fum_c0 + 2.0 na1_c0
reaction=model.reactions.get_by_id("RXN0906_c0")
reaction.gene_reaction_rule = "RSP_0910"

#Change GPR for udcpdp_c0 + h2o_c0 --> pi_c0 + h_c0 + udcpp_c0
reaction=model.reactions.get_by_id("RXN0581_c0")
reaction.gene_reaction_rule = "RSP_1150 or RSP_0119 or RSP_2161"

#Change GPR for 4per_c0 + nad_c0 <=> ohpb_c0 + h_c0 + nadh_c0
reaction=model.reactions.get_by_id("RXN1321_c0")
reaction.gene_reaction_rule = "Unknown"
```

```
#Change GPR for h2o_c0 + ahcys_c0 <=> hcys-L_c0 + adn_c0
reaction=model.reactions.get_by_id("RXN0150_c0")
reaction.gene_reaction_rule = "RSP_1514 or RSP_1516"

#Change GPR for fgam_c0 + h2o_c0 + atp_c0 + gln-L_c0 <=>
fpram_c0 + adp_c0 + h_c0 + glu-L_c0 + pi_c0
reaction=model.reactions.get_by_id("RXN0703_c0")
reaction.gene_reaction_rule = "RSP_2951"

#Make "atp_c0 + 12dgr_c0 <=> 12dag3p_c0 + h_c0 + adp_c0"
irreversible.
rxn=model.reactions.get_by_id("RXN0376_c0")
rxn.lower_bound = 0
rxn.upper_bound = 1000

#Add "12dag3p_c0 + h20_c0 <--> 12dgr_c0 + pi_c0"
reaction = Reaction('RXN1826_c0')
reaction.name = ''
reaction.subsystem = ''
reaction.lower_bound = -1000.  # This is the default
reaction.upper_bound = 1000.  # This is the default
reaction.objective_coefficient = 0. # this is the default
reaction.add_metabolites({model.metabolites.get_by_id("cpd11422_
c0"): -1.0,

model.metabolites.get_by_id("cpd00001_c0"): -1.0,

model.metabolites.get_by_id("cpd11423_c0"): 1.0,

model.metabolites.get_by_id("cpd00009_c0"): 1.0})

reaction.gene_reaction_rule = 'Unknown'

model.add_reaction(reaction)

#Change GPR for ppi_c0 + prbatp_c0 <=> prpp_c0 + atp_c0
reaction=model.reactions.get_by_id("RXN0432_c0")
reaction.gene_reaction_rule = "RSP_3549 and RSP_3550"

#Change GPR for ppi_c0 + prbatp_c0 <=> prpp_c0 + atp_c0
RSP_3549
reaction=model.reactions.get_by_id("RXN0073_c0")
reaction.gene_reaction_rule = "RSP_1808"

#Change GPR for cys-L_c0 + atp_c0 + trnacys_c0 --> cystrna_c0 +
amp_c0 + ppi_c0
```

```
reaction=model.reactions.get_by_id("RXN0061_c0")
reaction.gene_reaction_rule = "RSP_2722"

#Change GPR for trnaphe_c0 + phe-L_c0 + atp_c0 --> phetrna_c0 +
amp_c0 + ppi_c0
reaction=model.reactions.get_by_id("RXN0071_c0")
reaction.gene_reaction_rule = "RSP_1761 and RSP_1763"

#Remove s17bp_c0 + h2o_c0 --> s7p_c0 + pi_c0
rxn=model.reactions.get_by_id('RXN0113_c0')
rxn.delete()

#Change GPR for fdp_c0 + h2o_c0 --> f6p_c0 + pi_c0
reaction=model.reactions.get_by_id("RXN0556_c0")
reaction.gene_reaction_rule = "RSP_3266"

#Add reaction for consuming PAP; R. sphaeroides has gene
(RSP_1161) which is essential with Tn-Seq.

reaction = Reaction('RXN1825_c0')
reaction.name = ''
reaction.subsystem = ''
reaction.lower_bound = 0.  # This is the default
reaction.upper_bound = 1000.  # This is the default
reaction.objective_coefficient = 0. # this is the default
reaction.add_metabolites({model.metabolites.get_by_id("cpd00045_
c0"): -1.0,

model.metabolites.get_by_id("cpd00001_c0"): -1.0,

model.metabolites.get_by_id("cpd00018_c0"): 1.0,

model.metabolites.get_by_id("cpd00009_c0"): 1.0})

reaction.gene_reaction_rule = 'RSP_1161'

model.add_reaction(reaction)

#Delete oaa_c0 + h_c0 --> pyr_c0 + co2_c0
rxn=model.reactions.get_by_id('RXN1773_c0')
rxn.delete()

#TCA Cycle
#Make "acac_c0 + succoa_c0 <=> aacoa_c0 + succ_c0" irreversible
rxn=model.reactions.get_by_id('RXN0869_c0')
rxn.lower_bound = 0
rxn.upper_bound = 1000
```

```
#Set "aacoa_c0 + ac_c0 <=> acac_c0 + accoa_c0" to zero
rxn=model.reactions.get_by_id('RXN1772_c0')
rxn.lower_bound = 0
rxn.upper_bound = 0

#Make ump_c0 + ppi_c0 <=> ura_c0 + prpp_c0 operate in reverse
only
rxn=model.reactions.get_by_id('RXN0798_c0')
rxn.lower_bound = -1000
rxn.upper_bound = 0

#Delete h2o_c0 + uri_c0 <=> rib-D_c0 + ura_c0
rxn=model.reactions.get_by_id('RXN1719_c0')
rxn.delete()

#Delete h_c0 + h2o_c0 + csn_c0 --> nh4_c0 + ura_c0
rxn=model.reactions.get_by_id('RXN0801_c0')
rxn.delete()

#Change GPR for mn_c0 + h2o_c0 --> rnam_c0 + pi_c0
reaction=model.reactions.get_by_id("RXN0480_c0")
reaction.gene_reaction_rule = "RSP_2545"

#Change GPR for h2o_c0 + nicrnt_c0 --> pi_c0
reaction=model.reactions.get_by_id("RXN0481_c0")
reaction.gene_reaction_rule = "RSP_2545"

#Change GPR for amp_c0 + h2o_c0 --> adn_c0 + pi_c0
reaction=model.reactions.get_by_id("RXN0714_c0")
reaction.gene_reaction_rule = "RSP_2545"

#Change GPR for imp_c0 + h2o_c0 --> ins_c0 + pi_c0
reaction=model.reactions.get_by_id("RXN0715_c0")
reaction.gene_reaction_rule = "RSP_2545"

#Change GPR for h2o_c0 + gmp_c0 --> pi_c0 + gsn_c0
reaction=model.reactions.get_by_id("RXN0716_c0")
reaction.gene_reaction_rule = "RSP_2545"

#Change GPR for xmp_c0 + h2o_c0 --> xtsn_c0 + pi_c0
reaction=model.reactions.get_by_id("RXN0717_c0")
reaction.gene_reaction_rule = "RSP_2545"

#Change GPR for dgmp_c0 + h2o_c0 --> dgsn_c0 + pi_c0
reaction=model.reactions.get_by_id("RXN0718_c0")
reaction.gene_reaction_rule = "RSP_2545"
```

```
#Change GPR for damp_c0 + h2o_c0 --> pi_c0 + dad-2_c0
reaction=model.reactions.get_by_id("RXN0719_c0")
reaction.gene_reaction_rule = "RSP_2545"

#Change GPR for dcmp_c0 + h2o_c0 --> dcyt_c0 + pi_c0
reaction=model.reactions.get_by_id("RXN0784_c0")
reaction.gene_reaction_rule = "RSP_2545"

#Change GPR for atp_c0 + cmp_c0 --> adp_c0 + cdp_c0
reaction=model.reactions.get_by_id("RXN0792_c0")
reaction.gene_reaction_rule = "RSP_2705"

#Change GPR for h2o_c0 + atp_c0 + pi_p0 --> 2.0 pi_c0 + adp_c0 +
h_c0
reaction=model.reactions.get_by_id("RXN0919_c0")
reaction.gene_reaction_rule = " ((RSP_2601 and RSP_2602 and
RSP_2603 and RSP_2604) or (RSP_3968 and RSP_3967 and RSP_3966
and RSP_3964) or (RSP_3968 and RSP_3967 and RSP_3966 and
RSP_3965) or (RSP_3297 and RSP_3294 and RSP_3293 and RSP_3295)
or (RSP_3297 and RSP_3294 and RSP_3293 and RSP_3296) or
(RSP_2923 and RSP_2926 and RSP_2927 and RSP_2925) or (RSP_2923
and RSP_2926 and RSP_2927 and RSP_2924) or (RSP_2275 and
RSP_2277 and RSP_2276 and RSP_2274) or (RSP_2275 and RSP_2277
and RSP_2276 and RSP_2278) or (RSP_1290 and RSP_1291 and
RSP_1289 and RSP_1287) or (RSP_1290 and RSP_1291 and RSP_1289
and RSP_1288) or (RSP_0301 and RSP_0300 and RSP_0299 and
RSP_0297) or (RSP_0301 and RSP_0300 and RSP_0299 and RSP_0298))"

#Change GPR for 2.0 amet_c0 + cpppg3_c0 + 2.0 h_c0 --> pppg9_c0
+ 2.0 co2_c0 + 2.0 met-L_c0 + 2.0 dad-5_c0
reaction=model.reactions.get_by_id("RXN0631_c0")
reaction.gene_reaction_rule = "RSP_0317"

#Add Reactions for Anaerobic Vitamin B12 Synthesis
#Add pre2_c0 + nad_c0 --> scl_c0 + 2.0 h_c0 + nadh_c0

cpd03426_c0 =
Metabolite('cpd03426_c0',formula='',name='',compartment='c0')

reaction = Reaction('RXN1827_c0')
reaction.name = 'precorrin-2 dehydrogenase'
reaction.subsystem = ''
reaction.lower_bound = 0.  # This is the default
reaction.upper_bound = 1000.  # This is the default
reaction.objective_coefficient = 0. # this is the default
reaction.add_metabolites({model.metabolites.get_by_id("cpd01620_
```

```
c0"): -1.0,

model.metabolites.get_by_id("cpd00003_c0"): -1.0,
                          cpd03426_c0 : 1.0,

model.metabolites.get_by_id("cpd00004_c0"): 1.0,

model.metabolites.get_by_id("cpd00067_c0"): 2.0})

reaction.gene_reaction_rule = 'Unknown'

model.add_reaction(reaction)

#Add scl_c0 + cobalt2_c0 --> copre2_c0 + 2.0 h_c0

cpd08368_c0 =
Metabolite('cpd08368_c0',formula='',name='',compartment='c0')

reaction = Reaction('RXN1828_c0')
reaction.name = 'sirohydrochlorin cobaltochelatase'
reaction.subsystem = ''
reaction.lower_bound = 0.  # This is the default
reaction.upper_bound = 1000.  # This is the default
reaction.objective_coefficient = 0. # this is the default
reaction.add_metabolites({cpd03426_c0 : -1.0,

model.metabolites.get_by_id("cpd00149_c0"): -1.0,
                          cpd08368_c0: 1.0,

model.metabolites.get_by_id("cpd00067_c0"): 2.0})

reaction.gene_reaction_rule = 'Unknown'

model.add_reaction(reaction)

cpd17535_c0 =
Metabolite('cpd17535_c0',formula='',name='',compartment='c0')

#Add amet_c0 + copre2_c0 --> Cobalt-factor III_c0 + h_c0 +
ahcys_c0

reaction = Reaction('RXN1829_c0')
reaction.name = 'cobalt-sirohydrochlorin (C20)-methyltransferase
'
reaction.subsystem = ''
reaction.lower_bound = 0.  # This is the default
reaction.upper_bound = 1000.  # This is the default
```

```
reaction.objective_coefficient = 0. # this is the default
reaction.add_metabolites({cpd08368_c0 : -1.0,

model.metabolites.get_by_id("cpd00017_c0"): -1.0,
                          cpd17535_c0: 1.0,

model.metabolites.get_by_id("cpd00019_c0"): 1.0,

model.metabolites.get_by_id("cpd00067_c0"): 1.0})

reaction.gene_reaction_rule = 'RSP_2826'

model.add_reaction(reaction)

#Add fdxrd_c0 + Cobalt-factor III_c0 --> fdxox_c0 + copre3_c0

cpd08369_c0 =
Metabolite('cpd08369_c0',formula='',name='',compartment='c0')

reaction = Reaction('RXN1830_c0')
reaction.name = 'cobalt-precorrin 3 dehydrogenase'
reaction.subsystem = ''
reaction.lower_bound = 0.  # This is the default
reaction.upper_bound = 1000.  # This is the default
reaction.objective_coefficient = 0. # this is the default
reaction.add_metabolites({cpd17535_c0 : -1.0,

model.metabolites.get_by_id("cpd11620_c0"): -1.0,
                          cpd08369_c0: 1.0,

model.metabolites.get_by_id("cpd11621_c0"): 1.0})

reaction.gene_reaction_rule = 'Unknown'

model.add_reaction(reaction)

#Add amet_c0 + copre3_c0 --> copre4_c0 + ahcys_c0
#Cobalt-precorrin 3 + S-Adenosyl-L-methionine <=> Cobalt-
precorrin 4 + S-Adenosyl-L-homocysteine (RSP_2824)

cpd08370_c0 =
Metabolite('cpd08370_c0',formula='',name='',compartment='c0')

reaction = Reaction('RXN1831_c0')
reaction.name = 'precorrin 3 methyltransferase'
reaction.subsystem = ''
reaction.lower_bound = 0.  # This is the default
```

```
reaction.upper_bound = 1000.  # This is the default
reaction.objective_coefficient = 0. # this is the default
reaction.add_metabolites({cpd08369_c0 : -1.0,

model.metabolites.get_by_id("cpd00017_c0"): -1.0,
                          cpd08370_c0: 1.0,

model.metabolites.get_by_id("cpd00019_c0"): 1.0})

reaction.gene_reaction_rule = 'RSP_2824'

model.add_reaction(reaction)

#Add copre4_c0 + amet_c0 --> copre5_c0 + ahcys_c0
#Cobalt-precorrin 4 + S-Adenosyl-L-methionine <=> Cobalt-
precorrin 5A + S-Adenosyl-L-homocysteine

cpd08371_c0 =
Metabolite('cpd08371_c0',formula='',name='',compartment='c0')

reaction = Reaction('RXN1832_c0')
reaction.name = 'precorrin 4 methyltransferase'
reaction.subsystem = ''
reaction.lower_bound = 0.  # This is the default
reaction.upper_bound = 1000.  # This is the default
reaction.objective_coefficient = 0. # this is the default
reaction.add_metabolites({cpd08370_c0 : -1.0,

model.metabolites.get_by_id("cpd00017_c0"): -1.0,
                          cpd08371_c0: 1.0,

model.metabolites.get_by_id("cpd00019_c0"): 1.0})

reaction.gene_reaction_rule = 'RSP_2821'

model.add_reaction(reaction)

#Add h2o_c0 + copre5_c0 --> acald_c0 + 2.0 h_c0 + Cobalt-
precorrin 5B_c0
#cobalt-precorrin-5A + H2O = cobalt-precorrin-5B + acetaldehyde
+ 2 H+

cpd14960_c0 =
Metabolite('cpd14960_c0',formula='',name='',compartment='c0')

reaction = Reaction('RXN1833_c0')
reaction.name = 'cobalt-precorrin 5A acylhydrolase'
```

```
reaction.subsystem = ''
reaction.lower_bound = 0.  # This is the default
reaction.upper_bound = 1000.  # This is the default
reaction.objective_coefficient = 0. # this is the default
reaction.add_metabolites({cpd08371_c0 : -1.0,

model.metabolites.get_by_id("cpd00001_c0"): -1.0,
                          cpd14960_c0: 1.0,

model.metabolites.get_by_id("cpd00067_c0"): 2.0,

model.metabolites.get_by_id("cpd00071_c0"): 1.0})

reaction.gene_reaction_rule = 'RSP_2822'

model.add_reaction(reaction)

#Add "Cobalt-precorrin 5B_c0 + amet_c0 --> copre6_c0 + ahcys_c0"

cpd08372_c0 =
Metabolite('cpd08372_c0',formula='',name='',compartment='c0')

reaction = Reaction('RXN1834_c0')
reaction.name = 'cobalt-precorrin-5B (C1)-methyltransferase'
reaction.subsystem = ''
reaction.lower_bound = 0.  # This is the default
reaction.upper_bound = 1000.  # This is the default
reaction.objective_coefficient = 0. # this is the default
reaction.add_metabolites({cpd14960_c0 : -1.0,

model.metabolites.get_by_id("cpd00017_c0"): -1.0,
                          cpd08372_c0: 1.0,

model.metabolites.get_by_id("cpd00019_c0"): 1.0})

reaction.gene_reaction_rule = 'Unknown'

model.add_reaction(reaction)

#Add "copre6_c0 + nadh_c0 + h_c0 --> codhpre6_c0 + nad_c0"

cpd08373_c0 =
Metabolite('cpd08373_c0',formula='',name='',compartment='c0')

reaction = Reaction('RXN1835_c0')
reaction.name = 'precorrin-6A reductase'
reaction.subsystem = ''
```

```
reaction.lower_bound = 0.  # This is the default
reaction.upper_bound = 1000.  # This is the default
reaction.objective_coefficient = 0. # this is the default
reaction.add_metabolites({cpd08372_c0 : -1.0,

model.metabolites.get_by_id("cpd00004_c0"): -1.0,

model.metabolites.get_by_id("cpd00067_c0"): -1.0,
                          cpd08373_c0: 1.0,

model.metabolites.get_by_id("cpd00003_c0"): 1.0})

reaction.gene_reaction_rule = 'RSP_2825'

model.add_reaction(reaction)

#Add "amet_c0 + codhpre6_c0 --> ahcys_c0 + Cobalt-precorrin 7_c0
+ co2_c0"

cpd14961_c0 =
Metabolite('cpd14961_c0',formula='',name='',compartment='c0')

reaction = Reaction('RXN1836_c0')
reaction.name = 'cobalt-precorrin-6B (C15)-methyltransferase
[decarboxylating]'
reaction.subsystem = ''
reaction.lower_bound = 0.  # This is the default
reaction.upper_bound = 1000.  # This is the default
reaction.objective_coefficient = 0. # this is the default
reaction.add_metabolites({cpd08373_c0 : -1.0,

model.metabolites.get_by_id("cpd00017_c0"): -1.0,
                          cpd14961_c0: 1.0,

model.metabolites.get_by_id("cpd00019_c0"): 1.0,

model.metabolites.get_by_id("cpd00011_c0"): 1.0})

reaction.gene_reaction_rule = 'RSP_2823'

model.add_reaction(reaction)

#Add amet_c0 + Cobalt-precorrin 7_c0 --> ahcys_c0 + copre8_c0

cpd08375_c0 =
Metabolite('cpd08375_c0',formula='',name='',compartment='c0')
```

```
reaction = Reaction('RXN1837_c0')
reaction.name = 'cobalt-precorrin-7 (C5)-methyltransferase'
reaction.subsystem = ''
reaction.lower_bound = 0.  # This is the default
reaction.upper_bound = 1000.  # This is the default
reaction.objective_coefficient = 0. # this is the default
reaction.add_metabolites({cpd14961_c0 : -1.0,

model.metabolites.get_by_id("cpd00017_c0"): -1.0,
                          cpd08375_c0: 1.0,

model.metabolites.get_by_id("cpd00019_c0"): 1.0})

reaction.gene_reaction_rule = 'RSP_2823'

model.add_reaction(reaction)

#Add "copre8_c0 --> cobya_c0"

cpd03421_c0 =
Metabolite('cpd03421_c0',formula='',name='',compartment='c0')

reaction = Reaction('RXN1838_c0')
reaction.name = 'cobalt-precorrin-8 methylmutase'
reaction.subsystem = ''
reaction.lower_bound = 0.  # This is the default
reaction.upper_bound = 1000.  # This is the default
reaction.objective_coefficient = 0. # this is the default
reaction.add_metabolites({cpd08375_c0 : -1.0,
                          cpd03421_c0: 1.0})

reaction.gene_reaction_rule = 'RSP_6217'

model.add_reaction(reaction)

#Add "2.0 h2o_c0 + 2.0 gln-L_c0 + 2.0 atp_c0 + cobya_c0 -->
adcobdam_c0 + 2.0 glu-L_c0 + 2.0 pi_c0 + 2.0 adp_c0"

reaction = Reaction('RXN1839_c0')
reaction.name = 'cobyrinic acid a,c-diamide synthase'
reaction.subsystem = ''
reaction.lower_bound = 0.  # This is the default
reaction.upper_bound = 1000.  # This is the default
reaction.objective_coefficient = 0. # this is the default
reaction.add_metabolites({cpd03421_c0 : -1.0,

model.metabolites.get_by_id("cpd00002_c0") : -2.0,
```

```
model.metabolites.get_by_id("cpd00053_c0") : -2.0,

model.metabolites.get_by_id("cpd00001_c0") : -2.0,

model.metabolites.get_by_id("cpd03914_c0") : 1.0,

model.metabolites.get_by_id("cpd00008_c0") : 2.0,

model.metabolites.get_by_id("cpd00023_c0") : 2.0,

model.metabolites.get_by_id("cpd00009_c0") : 2.0})

reaction.gene_reaction_rule = 'RSP_3185'

model.add_reaction(reaction)

#Add anaerobic synthesis of 5,6 Dimethylbenzimidizole
#Add air_c0 + 4.0 h_c0 + amet_c0 --> ahcys_c0 + 3.0 h2o_c0 +
dmbzid_c0 + nh4_c0 + pi_c0

reaction = Reaction('RXN1840_c0')
reaction.name = ''
reaction.subsystem = ''
reaction.lower_bound = 0.  # This is the default
reaction.upper_bound = 1000.  # This is the default
reaction.objective_coefficient = 0. # this is the default
reaction.add_metabolites({model.metabolites.get_by_id("cpd02140_
c0") : -1.0,

model.metabolites.get_by_id("cpd00017_c0") : -1.0,

model.metabolites.get_by_id("cpd00067_c0") : -4.0,

model.metabolites.get_by_id("cpd01997_c0") : 1.0,

model.metabolites.get_by_id("cpd00019_c0") : 1.0,

model.metabolites.get_by_id("cpd00013_c0") : 1.0,

model.metabolites.get_by_id("cpd00009_c0") : 1.0,

model.metabolites.get_by_id("cpd00001_c0") : 3.0})

reaction.gene_reaction_rule = 'Unknown'

model.add_reaction(reaction)
```

```
#Anaerobic Vitamin B6 Metabolism

#Hydroxypyruvate <=> Glycolaldehyde + CO2

reaction = Reaction('RXN1841_c0')
reaction.name = 'hydroxypyruvate carboxy-lyase (glycolaldehyde-
forming)'
reaction.subsystem = ''
reaction.lower_bound = 0.  # This is the default
reaction.upper_bound = 1000.  # This is the default
reaction.objective_coefficient = 0. # this is the default
reaction.add_metabolites({model.metabolites.get_by_id("cpd00145_
c0") : -1.0,

model.metabolites.get_by_id("cpd00229_c0") : 1.0,

model.metabolites.get_by_id("cpd00011_c0") : 1.0})

reaction.gene_reaction_rule = 'Unknown'

model.add_reaction(reaction)

#Glycolaldehyde + 2-Oxobutanoate + Alanine <=> Pyridoxine

reaction = Reaction('RXN1842_c0')
reaction.name = ''
reaction.subsystem = ''
reaction.lower_bound = 0.  # This is the default
reaction.upper_bound = 1000.  # This is the default
reaction.objective_coefficient = 0. # this is the default
reaction.add_metabolites({model.metabolites.get_by_id("cpd00229_
c0") : -1.0,

model.metabolites.get_by_id("cpd00094_c0") : -1.0,

model.metabolites.get_by_id("cpd00117_c0") : -1.0,

model.metabolites.get_by_id("cpd00263_c0") : 1.0})

reaction.gene_reaction_rule = 'Unknown'

model.add_reaction(reaction)

#pyridoxine + NADP+ = pyridoxal + NADPH + H+

reaction = Reaction('RXN1843_c0')
```

```
reaction.name = ''
reaction.subsystem = ''
reaction.lower_bound = 0.  # This is the default
reaction.upper_bound = 1000.  # This is the default
reaction.objective_coefficient = 0. # this is the default
reaction.add_metabolites({model.metabolites.get_by_id("cpd00263_
c0") : -1.0,

model.metabolites.get_by_id("cpd00006_c0") : -1.0,

model.metabolites.get_by_id("cpd00215_c0") : 1.0,

model.metabolites.get_by_id("cpd00005_c0") : 1.0,

model.metabolites.get_by_id("cpd00067_c0") : 1.0})

reaction.gene_reaction_rule = 'Unknown'

model.add_reaction(reaction)

#Change GPR for 2.0 Photon_c0 + 2.0 h_c0 + q_c0 + 2.0 focytc_c0
<=> qh2_c0 + 2.0 ficytc_c0
reaction=model.reactions.get_by_id("RXN0620_c0")
reaction.gene_reaction_rule = "(RSP_0256 and RSP_0257)"

#Change GPR for Divinylprotochlorophyllide_c0 + nadph_c0 + h_c0
<=> Protochlorophyllide_c0 + nadp_c0
reaction=model.reactions.get_by_id("RXN0635_c0")
reaction.gene_reaction_rule = "(RSP_0280 or RSP_3070)"

#mg2_c0 + atp_c0 + h2o_c0 + ppp9_c0 <=> mppp9_c0 + pi_c0 + 2.0
h_c0 + adp_c0
reaction=model.reactions.get_by_id("RXN0623_c0")
reaction.gene_reaction_rule = "(RSP_0273 and RSP_0274)"

######################
print("Reactions: " + str(len(model.reactions)))
print("Metabolites: " + str(len(model.metabolites)))
print("Genes: " + str(len(model.genes)))

############################################################
#This section converts KBASE compound IDs to compound short
names.
############################################################

cmpd_table =
np.loadtxt('KBase_All_Compounds.txt',delimiter='\t',dtype=object
```

```
,skiprows=1,comments=None)

print(cmpd_table[0])

#This pulls in the forumla IDs
for m in model.metabolites:
    id = (m.id[:-3])
    for row in cmpd_table:
        if id == row[0]:
            formula = (row[5])
            m.formula = formula
    #print(m.id)
    #print(m.formula)

#This replaces the CPD##### with the abbreviation for making
flux maps with compound abbreviations
for m in model.metabolites:
    id = (m.id[:-3])
    for row in cmpd_table:
        if id == row[0]:
            m.notes = id
            abbrev = (row[2])
            m.id = abbrev+"_"+m.compartment

############################################################
#This section sets the media composition.
############################################################

Media = {'EX_cpd00001_e0':-1000, # EX_cpd00001 = H20;
         #'EX_cpd00007_e0':-1000, # EX_cpd00007 = O2;
         'EX_cpd00009_e0':-1000, # EX_cpd00009 = Phosphate;
         #'EX_cpd00011_e0':-1000, # EX_cpd00011 = CO2;
         'EX_cpd00013_e0':-1000, # EX_cpd00013 = NH3;
         #'EX_cpd00027_e0':-1, # EX_cpd00027 = Glucose
         'EX_cpd00030_e0':-1000, # EX_cpd00030 = Mn;
         'EX_cpd00034_e0':-1000, # EX_cpd00034 = Zn;
         'EX_cpd00036_e0':-1, # EX_cpd00036 = Succinate;
         'EX_cpd00048_e0':-1000, # EX_cpd00048 = Sulfate;
         'EX_cpd00058_e0':-1000, # EX_cpd00058 = Cu;
         'EX_cpd00063_e0':-1000, # EX_cpd00063 = Ca;
         'EX_cpd00067_e0':-1000, # EX_cpd00067 = H+;
         'EX_cpd00104_e0':-1000, # EX_cpd00104 = Biotin;
         'EX_cpd00149_e0':-1000, # EX_cpd00149 = Co;
         #'EX_cpd00154_e0':-1, #EX_cpd00154 = Xylose;
         'EX_cpd00205_e0':-1000, # EX_cpd00205 = K;
         'EX_cpd00218_e0':-1000, # EX_cpd00218 = Niacin;
         'EX_cpd00254_e0':-1000, # EX_cpd00254 = Mg;
```

```
        'EX_cpd00305_e0':-1000, # EX_cpd00305 = Thiamin;
        'EX_cpd00971_e0':-1000, # EX_cpd00971 = Na;
        'EX_cpd10515_e0':-1000, # EX_cpd10515 = Fe2+;
        'EX_cpd10516_e0':-1000, # EX_cpd10516 = Fe3+
        'EX_cpd31000_e0': -1000} # EX_cpd30001 = Photon #CHANGE
BIOMASS TO PHOTO BIOMASS



cobra.manipulation.modify.initialize_growth_medium(model,
the_medium=Media, external_boundary_compartment='e0',
reaction_lower_bound=0, reaction_upper_bound=1000 ,
irreversible=False, reactions_to_disable=None)

############################################################
#Sets the model objective function
############################################################

model.objective = "Biomass_Photo" # Chooose "Biomass_Photo" for
photo or "Biomass_Aero" for aero.
model.reactions.get_by_id("Biomass_Photo").objective_coefficient
= 1 # Chooose "Biomass_Photo" for photo or "Biomass_Aero" for
aero.

#########################################
#Set additional model contraints for Aerobic Growth
#########################################

"""#Set flux through RUBISCO to 0
rxn=model.reactions.get_by_id('RXN0109_c0')
rxn.lower_bound = 0
rxn.upper_bound = 0

#Turn off anaerobic porphyrin metabolism
#Set flux through "cpppg3_c0 + 2.0 amet_c0 + 2.0 h_c0 --> 2.0
co2_c0 + 2.0 dad-5_c0 + 2.0 met-L_c0 + pppg9_c0" to 0
rxn=model.reactions.get_by_id('RXN0631_c0')
rxn.lower_bound = 0
rxn.upper_bound = 0

#Turn off anaerobic Vitamin B12 metabolism
#Set flux through "nad_c0 + pre2_c0 --> scl_c0 + nadh_c0 + 2.0
h_c0" to 0
rxn=model.reactions.get_by_id('RXN1827_c0')
rxn.lower_bound = 0
rxn.upper_bound = 0
```

```
#Turn off anaerobic Vitamin B6 Metabolism
rxn=model.reactions.get_by_id('RXN1843_c0')
rxn.lower_bound = 0
rxn.upper_bound = 0"""

#########################################
#Set additional model contraints for Anaerobic Growth
#########################################

#Turn off oxygen export to prevent oxygen cycling in model
#Set flux through o2_p0 <=> o2_c0 to 0
rxn=model.reactions.get_by_id('RXN0949_c0')
rxn.lower_bound = 0
rxn.upper_bound = 0

#Set flux through o2_c0 + fmnh2_c0 --> e4p_c0 + h2o_c0 +
dmbzid_c0 to 0
rxn=model.reactions.get_by_id('RXN1318_c0')
rxn.lower_bound = 0
rxn.upper_bound = 0

#Set flux through 3.0 o2_c0 + pppg9_c0 --> 3.0 h2o2_c0 + ppp9_c0
to 0
rxn=model.reactions.get_by_id('RXN0661_c0')
rxn.lower_bound = 0
rxn.upper_bound = 0

#Set flux through o2_c0 + cpppg3_c0 + 2.0 h_c0 --> pppg9_c0 +
2.0 h2o_c0 + 2.0 co2_c0
rxn=model.reactions.get_by_id('RXN0630_c0')
rxn.lower_bound = 0
rxn.upper_bound = 0

#Mg-Protoporphyrin IX 13-monomethyl ester_c0 + h_c0 + nadph_c0 +
o2_c0 --> nadp_c0 + h2o_c0 + 13(1)-Hydroxy-Mg-protoporphyrin IX
13-monomethyl ester_c0
rxn=model.reactions.get_by_id('RXN0663_c0')
rxn.lower_bound = 0
rxn.upper_bound = 0

#Pyridoxine_5_phosphate_oxygen_oxidoreductase
rxn=model.reactions.get_by_id('RXN1063_c0')
rxn.lower_bound = 0
rxn.upper_bound = 0

#pydam_c0 + o2_c0 + h2o_c0 --> nh4_c0 + pydx_c0 + h2o2_c0
rxn=model.reactions.get_by_id('RXN1064_c0')
```

```
rxn.lower_bound = 0
rxn.upper_bound = 0

#8.0 h_c0 + o2_c0 + 4.0 focytc_c0 --> 4.0 h_p0 + 2.0 h2o_c0 +
4.0 ficytc_c0
rxn=model.reactions.get_by_id('RXN0515_c0')
rxn.lower_bound = 0
rxn.upper_bound = 0

#2oph_c0 + h_c0 + nadph_c0 + o2_c0 --> nadp_c0 + h2o_c0 +
2ohph_c0
rxn=model.reactions.get_by_id('RXN1020_c0')
rxn.lower_bound = 0
rxn.upper_bound = 0

#fdxrd_c0 + o2_c0 + hedacp_c0 + 2.0 h_c0 <=> cll_428_c0 +
fdxox_c0 + 2.0 h2o_c0
rxn=model.reactions.get_by_id('RXN1345_c0')
rxn.lower_bound = 0
rxn.upper_bound = 0

#2.0 gln-L_c0 + hgbyr_c0 + 2.0 atp_c0 + 2.0 h2o_c0 <=> 2.0 pi_c0
+ hgbam_c0 + 2.0 adp_c0 + 2.0 h_c0 + 2.0 glu-L_c0
rxn=model.reactions.get_by_id('RXN0648_c0')
rxn.lower_bound = 0
rxn.upper_bound = 1000

#########################################
#Constrain model with gene or reaction knockouts
#########################################

#rxn=model.reactions.get_by_id('RXN0511_c0')
#rxn.lower_bound = 0
#rxn.upper_bound = 0

#cobra.manipulation.delete_model_genes(model, ["RSP_2296"])

#########################################
#Run pFBA
#########################################

pFBA_solution =
cobra.flux_analysis.parsimonious.optimize_minimal_flux(model,alr
eady_irreversible=False)

print (model.solution.status)
print (model.solution.f)
```

```
print (model.objective)

#########################################
#Export model pFBA results to an Excel file
#########################################

#Create workbook that we will write the FBA flux results to.
workbook = xlsxwriter.Workbook('iRsp1140_Output_vSIS.xlsx')
worksheet = workbook.add_worksheet("pFBA_Results")
bold = workbook.add_format({'bold': 1})
worksheet.write('A1', 'REACTION', bold)
worksheet.write('B1', 'FLUX', bold)
worksheet.write('C1', 'NAME', bold)
worksheet.write('D1', 'EQUATION', bold)
worksheet.write('E1', 'GENES', bold)

#Write the flux results to the new excel file.
row = 1
col = 0

for i in model.solution.x_dict:
    if model.solution.x_dict[i] != float(0):
        worksheet.write_string(row, col, i)
        worksheet.write_number(row, col + 1,
model.solution.x_dict[i])
        for rxn in model.reactions:
            if rxn.id == i:
                worksheet.write_string(row, col + 2, rxn.name)
                worksheet.write_string(row, col + 3,
rxn.reaction)
                worksheet.write_string(row, col + 4,
rxn._gene_reaction_rule.decode('utf-8'))
        row +=1

### This section writes all of the reactions in the model to an
Excel workbook.
#Create workbook that we will write all of the model reactions
to.
worksheet2 = workbook.add_worksheet("All_Reactions")
worksheet2.write('A1', 'ID', bold)
worksheet2.write('B1', 'NAME', bold)
worksheet2.write('C1', 'REACTION', bold)
worksheet2.write('D1', 'GENE RULE', bold)
row = 1
col = 0
for rxn in model.reactions:
    worksheet2.write_string(row, col, rxn.id.decode('utf-8'))
```

```
    worksheet2.write_string(row, col + 1, 
rxn.name.decode('utf-8'))
    worksheet2.write_string(row, col + 2, 
rxn.reaction.decode('utf-8'))
    worksheet2.write_string(row, col + 3, 
rxn._gene_reaction_rule.decode('utf-8'))
    row +=1

workbook.close()


#########################################
#Save modified reconstruction as a JSON file
#########################################

cobra.io.save_json_model(model, "iRsp1140_vSISAero.json")

#########################################
#Run single-gene deletions to determine essential genes
#########################################

growth_rates, statuses = 
cobra.flux_analysis.single_gene_deletion(model)

#########################################
#Export knockout results to Excel
#########################################

workbook = 
xlsxwriter.Workbook('iRSP1140_vSIS_GeneKnockouts.xlsx')
worksheet = workbook.add_worksheet("KOs")
bold = workbook.add_format({'bold': 1})
worksheet.write('A1', 'GENE', bold)
worksheet.write('B1', 'BIOMASS_FLUX', bold)
worksheet.write('C1', 'ESSENTIAL?', bold)

#Write the flux results to the new excel file.
row = 1
col = 0
for i in growth_rates:
    worksheet.write_string(row, col, i)
    worksheet.write_number(row, col + 1, growth_rates[i])
    if growth_rates[i] <= 0.0001:
        worksheet.write_string(row, col+2, "YES")
    else:
        worksheet.write_string(row, col+2, "NO")
    row += 1
```

```
workbook.close()

##########################################
```
